# Phenotypic Characterization and Brain Structure Analysis of Calcium Channel Subunit α_2_δ-2 Mutant (Ducky) and α_2_δ Double Knockout Mice

**DOI:** 10.3389/fnsyn.2021.634412

**Published:** 2021-02-19

**Authors:** Stefanie M. Geisler, Ariane Benedetti, Clemens L. Schöpf, Christoph Schwarzer, Nadia Stefanova, Arnold Schwartz, Gerald J. Obermair

**Affiliations:** ^1^ Institute of Physiology, Medical University Innsbruck, Innsbruck, Austria; ^2^ Department of Pharmacology and Toxicology, Center for Molecular Biosciences, University of Innsbruck, Innsbruck, Austria; ^3^ Department of Pharmacology, Medical University Innsbruck, Innsbruck, Austria; ^4^ Division of Neurobiology, Department of Neurology, Medical University Innsbruck, Innsbruck, Austria; ^5^ Department of Pharmacology and Systems Physiology, College of Medicine, University of Cincinnati, Cincinnati, OH, United States; ^6^ Division Physiology, Karl Landsteiner University of Health Sciences, Krems an der Donau, Austria

**Keywords:** brain disease, CACNA2D, cortical lamination, over-grooming, stereology, voltage-gated calcium channels

## Abstract

Auxiliary α_2_δ subunits of voltage-gated calcium channels modulate channel trafficking, current properties, and synapse formation. Three of the four isoforms (α_2_δ-1, α_2_δ-2, and α_2_δ-3) are abundantly expressed in the brain; however, of the available knockout models, only α_2_δ-2 knockout or mutant mice display an obvious abnormal neurological phenotype. Thus, we hypothesize that the neuronal α_2_δ isoforms may have partially specific as well as redundant functions. To address this, we generated three distinct α_2_δ double knockout mouse models by crossbreeding single knockout (α_2_δ-1 and -3) or mutant (α_2_δ-2/ducky) mice. Here, we provide a first phenotypic description and brain structure analysis. We found that genotypic distribution of neonatal litters in distinct α_2_δ-1/-2, α_2_δ-1/-3, and α_2_δ-2/-3 breeding combinations did not conform to Mendel’s law, suggesting premature lethality of single and double knockout mice. Notably, high occurrences of infant mortality correlated with the absence of specific α_2_δ isoforms (α_2_Δ-2 > α_2_δ-1 > α_2_δ-3), and was particularly observed in cages with behaviorally abnormal parenting animals of α_2_δ-2/-3 cross-breedings. Juvenile α_2_δ-1/-2 and α_2_δ-2/-3 double knockout mice displayed a waddling gate similar to ducky mice. However, in contrast to ducky and α_2_δ-1/-3 double knockout animals, α_2_δ-1/-2 and α_2_δ-2/-3 double knockout mice showed a more severe disease progression and highly impaired development. The observed phenotypes within the individual mouse lines may be linked to differences in the volume of specific brain regions. Reduced cortical volume in ducky mice, for example, was associated with a progressively decreased space between neurons, suggesting a reduction of total synaptic connections. Taken together, our findings show that α_2_δ subunits differentially regulate premature survival, postnatal growth, brain development, and behavior, suggesting specific neuronal functions in health and disease.

## Introduction

In the central nervous system (CNS) the second messenger calcium regulates a variety of pivotal functions including neurotransmitter release, gene regulation, and synaptic plasticity (Nanou and Catterall, 2018). In healthy neurons, the entry of calcium is tightly controlled by voltage-gated calcium channels (VGCCs). Neuronal VGCCs are hetero-multimeric protein complexes consisting of a transmembrane pore-forming α_1_ subunit, which conducts Ca^2+^ upon membrane depolarization, and cytoplasmic β and extracellular α_2_δ subunits (Catterall, 2000; Zamponi et al., 2015). In vertebrates, four genes (Cacna2d1-4) encode four α_2_δ subunit isoforms (α_2_δ-1 to -4), which are post-translationally processed into highly glycosylated α_2_ and δ peptides linked to each other by disulfide bonds (De Jongh et al., 1990; Sandoval et al., 2004; Calderon-Rivera et al., 2012). The distinct isoforms share a protein sequence identity of approximately 60% (α_2_δ-3 vs. α_2_δ-4), 55% (α_2_δ-1 vs. α_2_δ-2), and 30% (α_2_δ-1/α_2_δ-2 vs. α_2_δ-3/α_2_δ-4; Klugbauer et al., 1999; Qin et al., 2002). Nevertheless, all α_2_δ subunit isoforms display a related topology with a rather similar domain structure (reviewed in Geisler et al., 2015; Dolphin, 2018).

Studies on the mRNA expression of the four α_2_δ subunit isoforms have revealed a partially differential and partially overlapping distribution in distinct organs including the heart, skeletal muscle, and pancreas (Ellis et al., 1988; Klugbauer et al., 1999; Gao et al., 2000; Arikkath and Campbell, 2003; Mastrolia et al., 2017). Notably, three out of four isoforms (α_2_δ-1, α_2_δ-2, and α_2_δ-3) are abundantly expressed in the brain (Klugbauer et al., 1999; Cole et al., 2005; Schlick et al., 2010; Geisler et al., 2019). α_2_δ-4 is the major isoform of retinal photoreceptor cells (Knoflach et al., 2013) and its expression in other CNS neurons seems negligible (Schlick et al., 2010). α_2_δ-1 has gained particular attention during the past years, as it contains a high-affinity binding site for the widely used anti-allodynic and anti-epileptic drugs gabapentin (Neurontin) and pregabalin (Lyrica; Gee et al., 1996; Gong et al., 2001; Fuller-Bicer et al., 2009).

Heterologous co-expression studies uncovered all α_2_δ subunit isoforms as potent modulators of calcium currents and membrane trafficking (Felix et al., 1997; Klugbauer et al., 1999; Hobom et al., 2000; Geisler et al., 2015; Dolphin, 2018). Beyond this principal role, isoform-specific synaptic functions have been proposed for α_2_δ-1 (Eroglu et al., 2009; Tong et al., 2017; Brockhaus et al., 2018; Chen et al., 2018; Risher et al., 2018), α_2_δ-2 (Fell et al., 2016; Tedeschi et al., 2016; Geisler et al., 2019), α_2_δ-3 (Pirone et al., 2014), and α_2_δ-4 (Wang et al., 2017; Kerov et al., 2018). Despite this increasing number of examples for α_2_δ-isoform specificity, it is unclear why distinct brain regions express three distinct isoforms (Cole et al., 2005; Schlick et al., 2010; Geisler et al., 2019). Moreover, all three neuronal isoforms could rescue a major defect in glutamatergic synapse formation observed in a cellular triple loss-of-function model (Schoepf et al., 2019).

Existing mouse models with spontaneous mutations and targeted deletions for all individual α_2_δ subunits enabled novel insights into their potential functional redundancy as well as specificity: deletion of α_2_δ-1 impaired synaptic NMDA receptor (NMDAR) recruitment, excitatory synaptogenesis, and spine morphology (Risher et al., 2018), and LTP-associated learning and memory (Zhou et al., 2018). Loss of full-length α_2_δ-2 in four distinct mouse strains with naturally occurring mutations [ducky: Barclay et al., 2001; Brodbeck et al., 2002; entla: Brill et al., 2004; and ducky(2J): Donato et al., 2006] and targeted deletions in Cacna2d2 (Ivanov et al., 2004) induced epilepsy, dyskinesia, cerebellar atrophy, and high mortality in juvenile mice. α_2_δ-3 knockout mice display altered pain processing (Neely et al., 2010), abnormal hearing (Pirone et al., 2014), anxiety-like behavior (Landmann et al., 2018a), and sensory cross-activation (Neely et al., 2010; Landmann et al., 2018b). Finally, distinct mouse strains with spontaneous mutations (Wycisk et al., 2006) and targeted deletions (Wang et al., 2017; Kerov et al., 2018) in the α_2_δ-4 isoform are associated with retinal degeneration and night blindness. Notably, of these existing mouse models, only α_2_δ-2 mutant mice display a highly decreased life span associated with severe neurological disease. This is insofar surprising as neurological disorders have been linked to aberrant α_2_δ subunit expression in humans: mutations in CACNA2D1 and CACNA2D2 with epilepsy (Chioza et al., 2009; Edvardson et al., 2013; Pippucci et al., 2013; Vergult et al., 2015; Butler et al., 2018), CACNA2D3 is a potential risk gene for autism spectrum disorders (Iossifov et al., 2012; De Rubeis et al., 2014), and all three genes with schizophrenia (Purcell et al., 2014; Moons et al., 2016; reviewed in Ablinger et al., 2020).

Thus, to provide novel insights into α_2_δ subunit specificity and redundancy we generated three double knockout mouse models by cross-breeding α_2_δ-1, α_2_δ-2, or α_2_δ-3 mouse strains. Here, we provide a phenotypic characterization and brain structure analysis of these newly established α_2_δ double knockout models. Moreover, we also included the characterization of adult ducky mice (8–10-weeks), as previous analyzes were restricted to young mice (~1 month). Our findings underpin the general importance of α_2_δ subunits for normal development and survival. However, we further show that loss of distinct combinations of two isoforms differentially affected postnatal growth and brain development, which was associated with neurological disease including gait abnormalities, repetitive behaviors, and the occurrence of seizure symptoms. Together, these data support the conclusion that α_2_δ subunits are critically involved in both, partially redundant and also isoform-specific functions.

## Materials and Methods

### Breeding and Genotyping Procedures

#### Generation and Breeding of α_2_δ Double Knockout Mice

Double knockout mice lacking different combinations of α_2_δ subunit isoforms (referred to as α_2_δ-1/-3, α_2_δ-1/-2, and α_2_δ-2/-3 double knockout mice) were generated by cross-breeding previously established conventional mouse models with targeted deletions or spontaneous mutations for individual α_2_δ subunits.

##### α_2_δ-1 Knockout Mouse (Referred to as α_2_δ-1^−/−^)

The α_2_δ-1 knockout mouse was generated by introducing a targeted insertion into exon 2 of the Cacna2d1 gene (Fuller-Bicer et al., 2009). The original strain was maintained in a C57BL/6 genetic background and knockout mice displayed a cardiovascular phenotype (Fuller-Bicer et al., 2009) as well as deficits in mechanical and cold sensitivity (Patel et al., 2013). For our breeding colony, mice heterozygous for α_2_δ-1 were kept in a mixed 129J × C57BL/6N background. Male knockout animals could not be used for breeding double knockout mice due to their reduced life span caused by their progressing diabetic phenotype (Mastrolia et al., 2017).

##### α_2_δ-2 Null Mouse (Referred to as α_2_δ-2 Mutant, α_2_δ-2^du/du^ or Ducky Mice)

The naturally occurring ducky mutation was formerly discovered in a breeding stock at the Jackson Laboratory (Bar Harbor, ME, USA; Snell, 1955). It represents a spontaneous recessive autosomal mutation that is linked to a genomic rearrangement within the Cacna2d2 gene resulting in loss of the full-length protein (Cacna2d2^du^). Previous studies showed that affected homozygous mice display growth retardation, an ataxic waddling gate, paroxysmal dyskinesia, and absence epilepsy, as well as dysgenesis of selective regions of the CNS, especially of hindbrain structures including the brainstem and cerebellum (Snell, 1955; Meier, 1968; Brodbeck et al., 2002). The original strain was purchased from the Jackson Laboratory (strain: TKDU/DnJ, #000575: Bar Harbor, ME, USA) and carried a spontaneous tail kink mutation (tk) in repulsion with the ducky mutation (du). We backcrossed the mice into a C57BL/6N background for more than eight generations to eliminate the tail kink mutation and select for the ducky allele. We provided the Jackson Laboratory with these mice (backcrossed for three generations) as the original stock was lost (strain: B6N; TKDU-Myo5ad Cacna2d2du/J, #012889: Bar Harbor, ME, USA).

##### α_2_δ-3 Knockout Mouse (Referred to as α_2_δ-3^−/−^)

The α_2_δ-3 knockout mouse was generated and characterized by Deltagen (strain: B6.129P2-Cacna2d3tm1Dgen; San Mateo, CA, USA; Neely et al., 2010). Knockout was obtained by targeted insertion of a bacterial LacZ cassette into exon 15 of the Cacna2d3 gene, and a deletion of 11 base pairs (bp 1,521 to base 1,531), enabling concomitant expression of β galactosidase under the endogenous promoter (last accessed in January 2021^1^). Previous studies showed that α_2_δ-3 knockout mice display hearing deficits (Pirone et al., 2014) as well as anxiety-like behavior (Landmann et al., 2018a). Mice for our breeding colony were provided by Jutta Engel (Saarland University, Germany) with the consent of the Jackson Laboratory (#005780: Bar Harbor, ME, USA), and backcrossed for more than eight generations into a C57BL/6N background.

α_2_δ-1/-2, α_2_δ-1/-3, and α_2_δ-2/-3 double knockout mice and littermate controls were obtained by cross-breeding single knockout or heterozygous mice as described below [(see Results section: “Generation of α_2_δ Double Knockout Mice”) and in Schoepf et al. (2019)]. Both α_2_δ-2 and α_2_δ-3 mice were backcrossed into a C57BL/6N background for more than 10 generations before double knockout breedings. Breedings for α_2_δ-1/-2 and α_2_δ-1/-3 double knockouts were maintained in a mixed 129J × C57BL/6N background (α_2_δ-1) repeatedly crossed with C57BL/6N (α_2_δ-2 or α_2_δ-3). Thus, progeny displayed either an agouti (129J) or black (C57BL/6N) fur color, the latter being primarily selected for breeding cages.

#### Animal Care and Husbandry

Animal procedures for wildtype BALB/c and α_2_δ mutant mice were performed at the Medical University Innsbruck following institutional guidelines that follow national and international laws and policies (European council directive for laboratory animals 2010/63/EU). The animal studies were reviewed and approved by the Austrian Federal Ministry of Education, Science and Research (formerly bmwfw), license numbers BMWFW-66.011/0113-WF/V/3b/2014 and BMWFW-66.011/0114-WF/V/3b/2014. Mouse numbers used for this project were regularly reported to the Austrian Federal Ministry of Education, Science, and Research. All mouse lines were maintained at the central animal facility in Innsbruck (ZVTA) under standard housing conditions with a temperature-and-humidity-controlled environment, food and water *ad libitum*, and a 12 h light/dark cycle. Generally, we tried to avoid unnecessary handling and resultant stress (see signs for stress and anxiety-related behavior below) by adapting distinct husbandry strategies for all or individual mouse lines as described below.

##### Breeding

Breeding cages normally consisted of one male and one female mouse, kept together for the entire breeding period. During the entire mating and breeding time, we preferentially monitored mice using undisturbed observation whenever applicable. Double knockout breeding cages were left completely undisturbed and cleaned/monitored by the researcher only. In addition to the standard cage equipment including wood bedding, nesting material, and polycarbonate houses, the environment of breeding cages was further enriched with cardboard houses (Ehret, Tulln, Austria). Whenever individual mice showed progressing signs of stress/anxiety-like behavior—obvious by head trembling, excessive grooming, and increased jumping/activity upon handling—we ensured the application of humane endpoints (α_2_δ-2/-3; see “Results” section). To increase the postnatal survival chance of litters from α_2_δ-2/-3 inter-crosses, pregnant females displaying anxiety-like behavior were kept together with BALB/c foster mothers.

##### Weaning

Offspring derived from α_2_δ-2 and α_2_δ-1/-3 breeding pairs was housed in the parental cage until weaning between postnatal days (P) 21–28. Thereafter, weanlings were kept in groups of same-sex littermates. Since the high mortality previously reported for α_2_δ-2 mutant mice relates at least to some extent to difficulties in obtaining food and water (Snell, 1955), dried and moistened chow, as well as water gels (HydroGel, H007-70015; ssniff Spezialdiäten, Germany), were placed on the bottom of the cage twice a week. Moreover, in addition to the standard cage equipment described above, the environment of weaned α_2_δ-2 mutant mice was further enriched with cardboard houses which also aided in accessing surplus food from wire bar lids at the top of the cage. In a previous study, we showed that the majority of α_2_δ-1/-2 and α_2_δ-2/-3 double knockout mice required the application of humane endpoints (Schoepf et al., 2019). The underlying cause was found to be most likely multifactorial, including infanticide, malnutrition associated with a poor general condition, and seizures linked to the loss of the α_2_δ-2 isoform (Meier, 1968; Barclay et al., 2001). Thus, the health condition of individual animals was monitored and graded according to general signs of well-being including activity, body posture, and grooming behavior^2^. Accordingly, some α_2_Δ-1/-2 and α_2_δ-2/-3 double knockout mice required the use of humane endpoints and experiments were done slightly before weaning age [between P17 and 21, referred to as juvenile (3–4-week-old) for simplicity] or at weaning age.

#### Genotyping of α_2_δ Mutant Mice

Mice were genotyped for the respective α_2_δ alleles at weaning age or when used for experiments. To this end, DNA was either extracted from 1 to 2 mm tail (until October 2014) or ear punch biopsies (after October 2014; according to BMWFW guidelines) by applying the HOTSHOT method (Truett et al., 2000). Two microliter of the resultant DNA solution was used as a template for PCR genotyping following the GoTaq Flexi protocol (Promega, Fitchburg, WI, USA) as described (Geisler et al., 2019). *α_2_δ-*1 g*enotyping*: genotyping for the Cacna2d1 gene was done by use of standard PCR conditions (annealing at 52°C for 30 s). Forward (F) and reverse (R) primers were: wildtype-F1: 5′-GAGCTTTCTTTCTTCTGATTCCAC-3′, mutant-F2: 5′-CTGCACGAGACTAGTGAGACG-3′, R: 5′-ACATTCTCAAGACTGTAGGCAGAG-3′. Expected band sizes were 346 bp for wildtype (α_2_δ-1^+^^/+^) and 635 bp for knockout (α_2_δ-1^–/–^) animals, respectively, and heterozygous mice showed both bands. *α_2_δ-*2 g*enotyping*: genotyping for the ducky mutation was adapted from Brodbeck et al. (2002) by use of standard PCR conditions (annealing at 56°C for 30 s). Primers F: 5′–ACCTATCAGGCAAAAGGACG-3′ and R: 5′-AGGGATGGTGATTGGTTGGA-3′ produced a fragment of 541 bp from a region duplicated in the ducky allele. Subsequent enzymatic digestion of the mutant allele with BspHI (New England Biolabs, Ipswich, MA, USA) and gel electrophoresis resulted in two fragments (286 and 273 bp) while the wildtype allele remained uncut. Heterozygous mice could be distinguished from α_2_δ-2 mutant mice according to the relative intensities of the double band. To confirm the genomic duplication of the Cacna2d2 gene in potential α_2_δ-2 null mice a previously established copy number (CN) qPCR assay was used (Schoepf et al., 2019). To this end, DNA was extracted from sacrificed putative knockout and littermate controls by incubating tissue biopsies at 55°C and 550 rpm in 250 μl Direct PCR Tail Lysis reagent (VWR, Radnor, PA, USA) containing 2.5 μl Protease K (20 mg/ml, Roche, Basel, Switzerland). Following overnight lysis, samples were heated to 85°C for 45 min at 550 rpm to inactivate Protease K, centrifuged at 16,800× *g* for 1 min and DNA content was measured using a NanoDrop 2000 Spectrophotometer (Thermo Fisher Scientific, Waltham, MA, USA). Samples were run in triplicates on a 7,500 fast real-time PCR machine (50 cycles). For each reaction 8 μl DNA (5 ng/μl), 1 μl FAM-dye labeled Cacna2d2 CN (ID: Mm00270662-cn) and 1 μl Vic-dye labeled transferrin receptor (Tfrc, #4458370) assay were added to 10 μl TaqMan Universal PCR Master Mix. All products were purchased from Thermo Fisher Scientific (Waltham, MA, USA; formerly Applied Biosystems). Relative gene expression of Cacna2d2 was calculated with the ΔΔCt-method (2^ΔΔCT^), were ΔCt was defined as Ct (gene) – Ct (Tfrc, housekeeping gene) and ΔΔCt as ΔCt (putative homozygous) – ΔCt (WT control). Thus, expected ratios between ducky and Tfrc alleles were 1 for wildtype samples (2 du alleles), 1.5 for heterozygous samples (3 du alleles), and 2 for homozygous ducky samples (4 du alleles). *α_2_δ-3 genotyping*: wildtype-F1: 5′–TAGAAAAGATGCACTGGTCACCAGG-3′, mutant-F2: 5′-GGGCCAGCTCATTCCTCCCACTCAT-3′, R: 5′–GCAGAAGGCACATTGCCATACTCAC-3′ (annealing at 63°C for 30 s. Expected band sizes were 183 bp for wildtype (α_2_δ-3^+^^/+^) and 331 bp for knockout (α_2_δ-3^−/−^) animals, respectively, and heterozygous mice showed both bands.

#### Genotype Distribution in Neonatal Litters

Breeding cages containing female mice in their late gestation period were examined once daily for litters *via* undisturbed observation when applicable. Cages containing BALB/c foster mothers were not included in this analysis to avoid potential bias caused by differences in maternal caretaking abilities. Pups were considered newly born when first found, giving a 0–24-h variability in actual age (P0–1). Because some of the new-born pups obtained from α_2_δ-2 and α_2_δ-2/-3 breeding combinations were required at P1–P2 for another project (Schoepf et al., 2019), they were immediately marked on the paws using green tattoo ink (Ketchum Manufacturing Inc., Brockville, ON, Canada) and genotyped as described above. Alternatively, the number of pups was counted when first found (P0–1), followed by genotyping at weaning age or when needed for experiments (defined by scientific or humane endpoints, see above). In this case, subsequent analysis of expected and observed neonatal genotype ratios was solely done on litters with complete numbers at weaning. While this approach may give biased results in that it underestimates total numbers, excessive handling stress was reduced in the behaviorally sensitive α_2_δ mutant parenting animals. Statistical analysis for neonatal genotype ratios was calculated using the Chi-square test.

### The General Assessment of Behavioral Phenotypes

#### Pre-weaning Development

Initial observations on phenotypes of α_2_δ-1/-2, α_2_δ-1/-3, and α_2_δ-2/-3 double knockout pups were done once weekly when cleaning cages to reduce handling stress and increase survival chances of double knockout animals. General health was assessed by examining the grooming state of fur, posture, and responsiveness to handling. Since previous studies showed that ducky mice display ataxia and absence epilepsy (Snell, 1955; Meier, 1968; Brodbeck et al., 2002), we evaluated during handling if double knockout mice show symptoms which are typically associated with ataxic and epileptic conditions (wide based gait, whisker trembling during the behavioral arrest, loss of balance, falling to the side, and behavioral immobility; Ding et al., 2017; Van Erum et al., 2019).

#### Behavior in Breeding Pairs

It has been previously reported that α_2_δ-3 single knockout mice display an increased anxiety-like behavior (Landmann et al., 2018a), and CACNA2D3 is mentioned as a potential risk gene for autism spectrum disorders (Iossifov et al., 2012; De Rubeis et al., 2014). During weekly cleaning of cages we therefore monitored α_2_δ-1/-3 and α_2_δ-2/-3 breeding pairs for obvious signs of stress, anxiety, and repetitive behavior (e.g., excessive grooming, head tremble, hesitant behavior when opening the cage, and increased jumping/activity upon handling; Bolivar et al., 2007; Kalueff et al., 2016; Landmann et al., 2018a; Lee et al., 2018).

### Neuroanatomical Studies

#### Brain to Bodyweight Ratios

Body weights of juvenile or adult male mice were measured and subsequently mice were killed by CO_2_, decapitated, and the whole brains were quickly removed from the skull and immediately weighted. Samples comprised anterior tissue starting from the olfactory bulb to the posterior brainstem including the medulla. The spinal cord was cut off at the cerebellum. The relative weight of brain, body, and brain to body ratios of single and double knockout mice were calculated as a percentage of control.

#### Brain Tissue Preparation

Fresh whole brains of single/double knockouts and respective controls were removed from the skull as described above. To obtain sagittal sections, hemispheres were separated with a cut along the midline and placed medial side down on a flat piece of thin acryl glass (Geisler et al., 2019). Subsequently, mounted hemispheres were submerged for 1 min in 2-methylbutan (Carl Roth, Karlsruhe, Germany) cooled to −50°C. Frozen samples were stored in sealed vials at −80°C until further processing and transferred to −20°C 1 day before sectioning. Brain samples were mounted on a tissue holder using Tissue-Tek^®^ O.C.T.^TM^ Compound (A. Hartenstein, Würzburg, Germany). Consecutive sections (20 μm) of one hemisphere were obtained with a cryotome (NX50: Histocom, Vienna, Austria), collected on polysine coated glass slides (Lactan, Graz, Austria), and stored at −20°C until further use.

#### Nissl Staining and Volumetric Analysis

Nissl staining of every 15th slide was performed as described previously (Paxinos and Franklin, 2012) with some modifications. Briefly, sections were air-dried at room temperature (RT) for 15 min and fixed with freshly prepared cold 4% paraformaldehyde (pF) diluted in 1× phosphate-buffered saline (PBS, pH 7.4) for 10 min. After dipping slides in 1× PBS and Milli-Q (MQ) water, sections were dehydrated by an ascending ethanol series, followed by immersion in *n*-butyl acetate (Roth, Germany) for 10 min. Thereafter, samples were rehydrated by a descending ethanol series ending in MQ water. Nissl staining was performed for 20 min *via* incubation in a staining solution consisting of 0.5% cresyl violet acetate (Sigma–Aldrich, St. Louis, MO, USA), three parts MQ water, 1.7 parts 1 M acetic acid, and 0.3 parts sodium acetate. Staining was stopped by shortly immersing sections in MQ water and excess solution was removed *via* an ascending ethanol series followed by clearing with *n*-butyl acetate. The slides were mounted with Eukitt (Christine Gröpl, Tulln, Austria) and air-dried for subsequent volumetric analysis. Representative images of distinct brain regions were recorded with a BX53 microscope (Olympus, Tokyo, Japan) equipped with an SC100 color-camera (Olympus, Tokyo, Japan) using a 10× 0.40 NA objective. 8-bit panorama pictures were created by scanning specimens with a 4× 0.16 NA objective and using the manual multiple image alignment (MIA) function in cellSens Dimension software (Olympus, Tokyo, Japan).

Volumetric analysis of individual brain regions of interest (ROIs) was obtained with a Nikon Eclipse E800 microscope equipped with a Nikon camera DXM1200 and a Stereo Investigator Software driving a motorized stage (Micro Bright Field Europe, Magdeburg, Germany). The first section analyzed comprised the hippocampus and corpus callosum (~0.225 mm lateral to Bregma), and the last slide included the external capsule (~3.725 mm lateral to Bregma). Thus, 7–11 sections were analyzed per animal by an experimenter blinded to the genotypes. To select brain structures of interest we used the following criteria: (1) clearly outlined on Nissl-stained sections; and/or (2) previously shown to express brain α_2_δ subunit isoforms (α_2_δ-1, α_2_δ-2, and α_2_δ-3; Cole et al., 2005; Schlick et al., 2010); and/or (3) abnormal α_2_δ expression associated with consequences on structure or function (Brodbeck et al., 2002; Landmann et al., 2018b). Borders of the individual regions of interest were delineated according to the Mouse Brain Atlas (Paxinos and Franklin, 2012) and Redwine et al. (2003) as follows: cerebellum (rostral border: flocculus, middle cerebellar peduncle, and central lobule two—ventral border: dorsal to the fourth ventricle); corpus callosum (gray-white matter border); hippocampus (gray/white matter border with the fimbria/corpus callosum); whole hemisphere (rostral border: excluding olfactory bulbs—caudal border: medulla/spinal cord boundary at the most caudal point of the cerebellum); neocortex (dorsal border: corpus callosum—rostral border: rhinal fissure). The counter tracer option of the software was used to outline and measure the distinct ROIs on each slide. Subsequently, volumes were calculated according to the Cavalieri principle (Glaser and Glaser, 2000) by multiplying the sum of the areas with the uniform distance between the sections (15 × 20 μm).

#### Antibody Characterization

Cortical lamination was analyzed using well-established markers for transcription factors specifically expressed in distinct cortical layers (Nieto et al., 2004; Hevner, 2007; Molyneaux et al., 2007). Information on primary antibodies, which have been published and validated previously as described below, is summarized in Supplementary Table 1. The chicken ovalbumin upstream promoter transcription factor-interacting protein 2 antibody (Ctip2, also known as Bcl11b; amino acid residues 1–150: MSRRKQGNPQHLSQRELITPEADHVEAAILEEDEGLEIEEPSGLGLMVGG) detects two bands at about 120 kD on Western blots prepared from Jurkat cell lysates (T-cell line), possibly representing two CTIP2 isoforms (Senawong et al., 2003). Immunofluorescence analysis performed in the present study displayed immunopositive neurons in cortex layers V and VI, as well as hippocampus and striatum. Thus, the staining patterns reported here are in accordance with numerous studies using the same antibody on mouse brain sections (Arlotta et al., 2005; Chen et al., 2005; Huang et al., 2012; Betancourt et al., 2014; Chang et al., 2018).

The Homeobox protein Cut Like 1 antibody (Cux1, also known as Cutl1 or CDP; amino acid residues 1111–1332, C-terminal) detects the full-length 200-kDa protein (p200) as well as several truncated isoforms (p55 and p75) on Western blots prepared from nuclear extracts isolated from testes (Kroll et al., 2011). Moreover, a band of 200-kDa was detected in whole-cell protein extracts isolated from lungs of wildtype, but not of Cux1 knockout mice (Luong et al., 2002). In the present study, immunopositive neurons were identified in cortex layers II–IV, replicating staining patterns shown previously for this antibody (Jaitner et al., 2016; Abdurakhmanova et al., 2017; Chang et al., 2018).

The T-box brain 1 antibody (Tbr1, to amino acid residues 50–150: SPLKKITRGMTNQSDTDNFPDSKDSPGDVQRSKLSPVLDGVSELRHSFDGS) was previously validated for chromatin immunoprecipitation (Chip) on embryonic mouse cortices, further revealing specific binding to the deep-layer transcription factor Fezf2 (McKenna et al., 2011). Immunofluorescence analysis performed in the present study displayed immunopositive neurons in cortex layer II–IV, V, and VI, thus replicating the staining pattern reported in previous studies using the same antibody on mouse brain sections (Favero et al., 2013; Betancourt et al., 2014).

Primary antibodies were detected by fluorochrome-conjugated secondary goat-anti-rabbit Alexa Fluor 488 (1:4,000; Thermo Fisher Scientific, Waltham, MA, USA; Cat# A-11094, RRID:AB_221544) and goat-anti-rat Alexa Fluor 594 (1:4,000; Thermo Fisher Scientific, Waltham, MA, USA; Cat# A-11007, RRID: AB_10561522). The specificity of secondary antibodies was verified on cryosections by omitting primary antibody incubation, which gave no signal.

#### Immunohistochemistry and Cortex Analysis

Consecutive slides of sagittal cryosections obtained from α_2_δ-2 mutant (α_2_δ-2^du/du^) and control (α_2_δ-2^+^^/+^) mice were processed for immunohistochemistry as follows: brain slices were air-dried at RT for 15 min, surrounded with a hydrophobic liquid barrier (Roti^®^-Liquid Barrier Marker, colorless; Carl Roth, Karlsruhe, Germany) and fixed in 4% pF/4% sucrose in PBS for 5 min. Following washing with three changes of PBS and permeabilization in PBS containing 0.2% bovine serum albumin (BSA) and 0.2% Triton X-100 (PBS/BSA/Triton) for 5 min, samples were incubated in blocking solution (5% normal goat serum in PBS/BSA/Triton) for 2 h. Thereafter, slides were incubated in a blocking buffer containing the following combinations of primary antibodies (Supplementary Table 1): rat-anti-Ctip2 with rabbit-anti-Cux1 or rat-anti-Ctip2 and rabbit-anti-Tbr1, applied at 4°C overnight. After three subsequent washes in PBS/BSA/Triton for 10 min, primary antibodies were detected by fluorochrome-conjugated secondary goat-anti-rabbit Alexa Fluor 488 and goat-anti-rat Alexa Fluor 594, incubated for 2 h (diluted in blocking solution). Following three repeated washes with PBS/BSA/Triton for 30 min, cell nuclei were counterstained with Höchst33342 (1:10,000; #B2261: Sigma–Aldrich, St. Louis, MO, USA) for 5 min. Finally, slides were rinsed several times with PBS and MilliQ water, mounted with Vectashield (adult; #H-100: Szabo-Scandic, Vienna, Austria) or Fluoromount-G (juvenile; #0100-01: SouthernBiotech, Birmingham, AL, USA) and sealed with nail polish. All steps were done at RT except primary antibody incubation. For all subsequent analyzes described below, anatomically matched sections of α_2_δ-2 mutant mice and control animals were analyzed by an experimenter blind to the genotype.

***Cortical Length***. 8-bit panorama pictures were recorded with a BX53 microscope (Olympus, Tokyo, Japan) equipped with a cooled CCD camera (XM10, Olympus, Tokyo, Japan) as follows: specimens counterstained with Höchst were scanned with a 4× 0.16 NA objective lens. The MIA function in cellSens Dimension software (Olympus, Tokyo, Japan) was applied for image stitching to comprise the entire cortex in one image. The anteroposterior distance was measured from olfactory bulb/frontal cortex boundary (rhinal fissure) to posterior cortex/superior colliculus boundary (Mairet-Coello et al., 2012) using MetaMorph software (Molecular Devices, Sunnyvale, CA, USA). Measurements of two to four consecutive sections per brain were averaged in MS excel (total of two to four ROIs per sample), and two (adult) and four (juvenile) brains per genotype were analyzed (total of 4–16 ROIs per genotype).

***Cortical and Laminar Thickness***. 14-bit color images from triple fluorescence-labeled sections were acquired from Ctip2 (red; layer V), Cux1 (green; layer II–IV), or Tbr1 (green; layer VI), and Höchst (blue) channels using a BX53 microscope (Olympus, Tokyo, Japan) equipped with an SC100 color-camera (Olympus, Tokyo, Japan) and a 10× 0.40 NA objective lense. After the acquisition, corresponding Ctip, Cux/Tbr, and Höchst images were superimposed in Adobe Photoshop CS6 and analyzed in MetaMorph (Molecular Devices, Sunnyvale, CA, USA) and MS Excel. To this end, whole cortical thickness (layer I–VI) and laminar thickness (layer I, II–IV, V, and VI) were measured at the level of somatosensory cortex at three anteroposterior positions randomly selected within a region spanning from the anterior part of the lateral ventricle to the rostral hippocampus. two samples (adult; wildtype and knockout) were excluded from the analysis of laminar thickness, as they were not at a comparable cortical level. Three measurements per section and two to four consecutive sections per brain were averaged (total of 6–12 ROIs per sample), and two to three (adult) and four (juvenile) brains per genotype were analyzed (total of 12–48 ROIs per genotype). Finally, the absolute laminar thickness of individual layers of α_2_δ-2 mutant mice was calculated as the percentage change to control.

***Cell Density***. Calculation of cell densities was done using ImageJ software (NIH^2^; Schneider et al., 2012) as follows: Ctip (red), Tbr (green), and Höchst (blue) images were superimposed and a region comprising the entire cortical area was drawn, as well as a selection for background subtraction. Subsequently, the individual color images were thresholded (Image > Adjust > Threshold) to solely include cells positive for the respective marker. Background mean intensity was measured in distinct channels and the ROI surrounding the cortex was transferred to the thresholded Höchst image. The watershed function (Process > Binary > Watershed) was used to separate single cell nuclei. Thus, using the “analyze particle function,” thresholded cells exclusively within the cortical area were selected automatically as ROIs and counted (parameters: particle size: 0-infinity, excluding edges). ROIs were transferred to the Ctip/Tbr channel images and individual mean gray value intensities were measured in all three channels and further analyzed in MS Excel. Background subtractions for distinct channels were applied, providing the absolute number of positively stained cells. Cell numbers were then divided by the cortex area (mm^2^) to quantify the cellular density as the number of cells per mm^2^ cortex. The proportion of the following four categories was calculated as the percentage of Höchst^+^ cells (total cells = neurons and non-neuronal cells): (1) Ctip^–^/Tbr^–^/Höchst^+^ (layer I–IV neurons and non-neuronal cells); (2) Ctip^+^/Tbr^+^/Höchst^+^ (layer V–VI neurons); (3) Ctip^+^/Tbr^–^/Höchst^+^ (layer V neurons); (4) Ctip^–^/Tbr^+^/Höchst^+^ (layer VI neurons). Measurements of 2-4 consecutive sections per brain were averaged in MS excel (total of two to four ROIs per sample), and two (adult) and four (juvenile) brains per genotype were analyzed (total of 12–48 ROIs per genotype).

### Experimental Design and Statistical Analysis

According to the 3R principle, the minimum number of mice necessary for a statistical representative analysis was used. Humane endpoints and resultant experimental ages were chosen for the individual mouse lines according to the severity of the phenotype. Thus, research was primarily conducted on either juvenile (3–4-week-old; ducky, α_2_δ-1/-2 and α_2_δ-2/-3) or adult mice (8–13-week-old; ducky and α_2_δ-1/-3). The utilized controls were wildtype, heterozygous, or single knockout for a given α_2_δ isoform and included in most cases littermates or age-matched individuals. Mouse numbers, genotypes, and ages used for individual experiments are given in the figure legends. Where indicated, investigators were blinded during experiments and analyzes (see respective sections above). Data are depicted either as bar graphs showing means of mice ± SEM, or dot plots representing values of individual mice (dots) and means (line) ± SEM. *N*-numbers to calculate SEMs were given by the number of animals used. Before statistical analysis, the normality of data sets was evaluated with histograms (Sigma Plot, Systat Software GmbH, Erkrath, Germany). Significance levels (*p*-values) are presented in the respective figure legends or tables. *p*-values were calculated using an unpaired *t*-test or ANOVA with Holm–Sidak *posthoc* analysis (>2 groups). For the data presented in Figure 4, volumes of individual brain regions between controls and mutants were first compared using unpaired *t*-tests. Subsequently, *p-values* of all analyzed brain regions were manually corrected for multiple comparisons using Holm–Sidak *posthoc* adjustment (Supplementary Table 2). Data and graphs were organized and analyzed using MS Excel, Graph Pad Prism 6 (GraphPad Software, La Jolla, CA, USA), and Sigma Plot (Systat Software GmbH, Erkrath, Germany). Figures were assembled in Adobe Photoshop CS6 and linear adjustments were done to correct black level and contrast.

**Figure 1:**
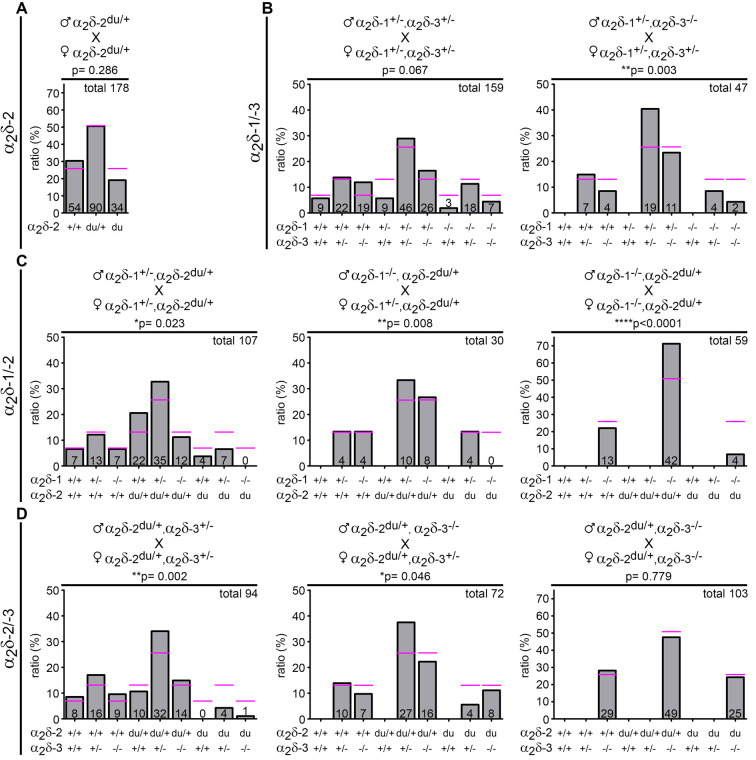
Genotype distribution is altered in neonatal litters bred from distinct α_2_δ inter-crosses. Expected (magenta lines) and observed (bar graphs) genotypes of neonatal offspring (P0–1) obtained by crossbreeding different α_2_δ mutant or single knockout mice (parental genotypes are shown above respective graphs). Symbols indicate either wildtype (+) or mutated (−) α_2_δ-1, α_2_δ-2, or α_2_δ-3 alleles. The absolute number of pups is displayed on the bars and the total amount of analyzed animals is depicted on the upper right side of each graph (confer Table 1 for further information on the number of analyzed litters and mean litter size). While the observed genotype ratio in litters was close to expected values when crossbreeding heterozygous α_2_δ-2 mutant mice **(A)** the frequency of distinct α_2_δ single knockout and double knockout mice was below expected ratios in α_2_δ-1/-3 **(B)** α_2_δ-1/-2 **(C)** and α_2_δ-2/-3 **(D)** inter-crosses. *Statistics*: Chi-square test: **(A)** $\chi_{\left(\text{2}\right)}^{\text{2}}$ = 2.5; **(B)** left: $\chi_{\left(\text{8}\right)}^{\text{2}}$ = 14.6, right: $\chi_{\left(\text{5}\right)}^{\text{2}}$ = 18.1; **(C)** left: $\chi_{\left(\text{8}\right)}^{\text{2}}$ = 17.8, middle: $\chi_{\left(\text{5}\right)}^{\text{2}}$ = 15.6, right: $\chi_{\left(\text{2}\right)}^{\text{2}}$ = 22.6; **(D)** left: $\chi_{\left(\text{8}\right)}^{\text{2}}$ = 24.2, middle: $\chi_{\left(\text{5}\right)}^{\text{2}}$ = 11.3, right: $\chi_{\left(\text{2}\right)}^{\text{2}}$ = 0.5. Exact *p-values* are given in the respective graphs. Asterisks in graphs indicate significance levels: *p < 0.05, **p < 0.01, ****p < 0.0001.

**Figure 2:**
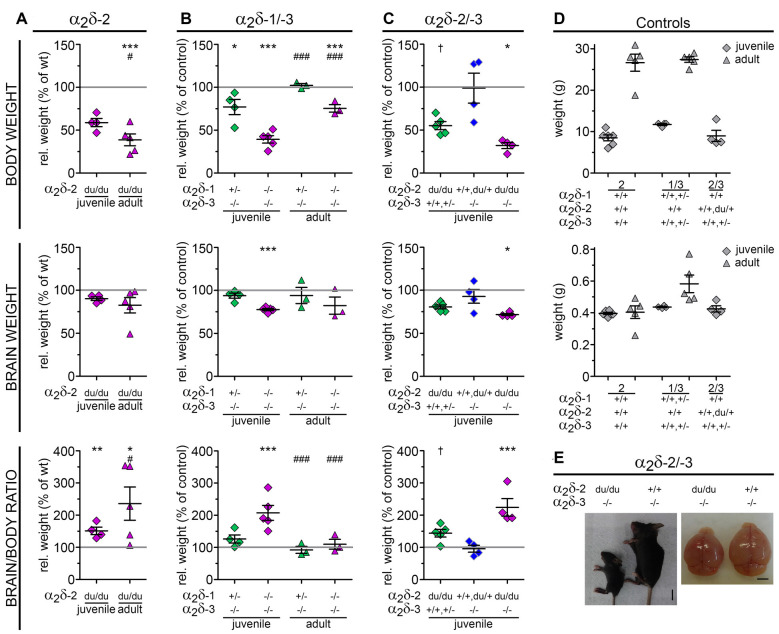
Loss of distinct α_2_δ subunits causes impaired development. Relative body weight, brain weight and brain/body ratios calculated as percentage of controls (gray line and raw values in **D**) as a measure for proper development of α_2_δ-2 mutant mouse ducky **(A)** and α_2_δ-1/-3 **(B)** or α_2_δ-2/-3 **(C)** double knockout mice. Juvenile (3-4-week-old) or adult mice (8–13-week-old) are depicted with squares or triangles, respectively. In all three mouse models juvenile mice showed a highly decreased body weight together with a moderately reduced brain size resulting in 1.5-fold (**A**, ducky), 2-fold (**B**, α_2_δ-1/-3), or 2.2-fold (**C**, α_2_δ-2/-3) higher brain/body ratios compared to controls. The magnitude of this effect varied with age: while brain/body ratios were normalized to control levels in adult α_2_δ-1/-3 double knockout mice **(B)** a relatively mild increase in body weight during adulthood together with a moderately reduced brain size resulted in even more elevated brain/body ratios in ducky mice (**A**, 2.4-fold). Exemplary images of a α_2_δ-2/-3 double knockout mouse (left) at P21, depicting the remarkably smaller body and brain size compared to its α_2_δ-3 single knockout littermate **(E)**. Values for individual animals (dots) and means (line) ± SEM are shown. *N-numbers*: **(A)** wildtype controls: six (juvenile) and five (adult), ducky mutant: four (juvenile) and five (adult); **(B)** wildtype or heterozygous controls: four (juvenile) and five (adult), α_2_δ-3 knockout: four (juvenile) and three (adult), α_2_δ-1/-3 double knockout: five (juvenile) and three (adult); **(C)** wildtype or heterozygous controls: 4, α_2_δ-2 knockout: 5, α_2_δ-3 knockout: 4, α_2_δ-2/-3 double knockout: 4. *Statistics*: two-way ANOVA with Holm–Sidak *posthoc* analysis: body weight: genotype: *F*_(1,16)_ = 45.8, *p* < 0.001, age: *F*_(1,16)_ = 64, *p* < 0.001, genotype × age: *F*_(1,16)_ = 19.2, *p* < 0.001; brain weight: genotype: *F*_(1,16)_ = 3.9, *p* = 0.064, age: *F*_(1,16)_ = 0.1, *p* = 0.7, genotype × age: *F*_(1,16)_ = 0.3, *p* = 0.6; Brain/body ratio: genotype: *F*_(1,16)_ = 18.6, *p* < 0.001, age: *F*_(1,16)_ = 39.8, *p* < 0.001, genotype × age: *F*_(1,16)_ = 0.1, *p* = 0.8; **(B)** two-way ANOVA with Holm–Sidak *posthoc* analysis: body weight: genotype: *F*_(2,18)_ = 50.7, *p* < 0.001, age: *F*_(2,18)_ = 764.5, *p* < 0.001, genotype × age: *F*_(2,18)_ = 2.8, *p* = 0.09; brain weight: genotype: *F*_(2,18)_ = 5.9, *p* = 0.01, age: *F*_(2,18)_ = 28.2, *p* < 0.001, genotype × age: *F*_(2,18)_ = 0.1, *p* = 0.9; Brain/body ratio: genotype: *F*_(2,18)_ = 9.2, *p* = 0.002, age: *F*_(2,18)_ = 115.5, *p* < 0.001, genotype × age: *F*_(2,18)_ = 5.0, *p* = 0.02; **(C)** one-way ANOVA with Holm–Sidak *posthoc* analysis: body weight: *F*_(3,11)_ = 6.6, *p* = 0.008, *post hoc*: ^†^*p* = 0.053 between α_2_δ-2 single knockout and control, *p < 0.05 between α_2_δ-2/-3 double knockout and α_2_δ-3 single knockout/control; brain weight: *F*_(3,11)_ = 5.8, *p* = 0.01, *posthoc*: ^†^*p* = 0.07 between α_2_δ-2 single knockout and control, *p = 0.015 between α_2_δ-2/-3 double knockout and control; brain/body ratio: *F*_(3,11)_ = 11.5, *p* = 0.001, *posthoc*: ***p = 0.001 between α_2_δ-2/-3 double knockout and control, **p = 0.002 between α_2_δ-2/-3 double knockout and α_2_δ-3 single knockout, *p = 0.012 between α_2_δ-2/-3 double knockout and α_2_δ-2 single knockout. Symbols in graphs indicate significance levels of factor genotype within (*), or factor age (^#^): ^†^*p* < 0.07, ^*/#^*p* < 0.05, **p < 0.01, ^***/###^*p* < 0.001.

**Figure 3:**
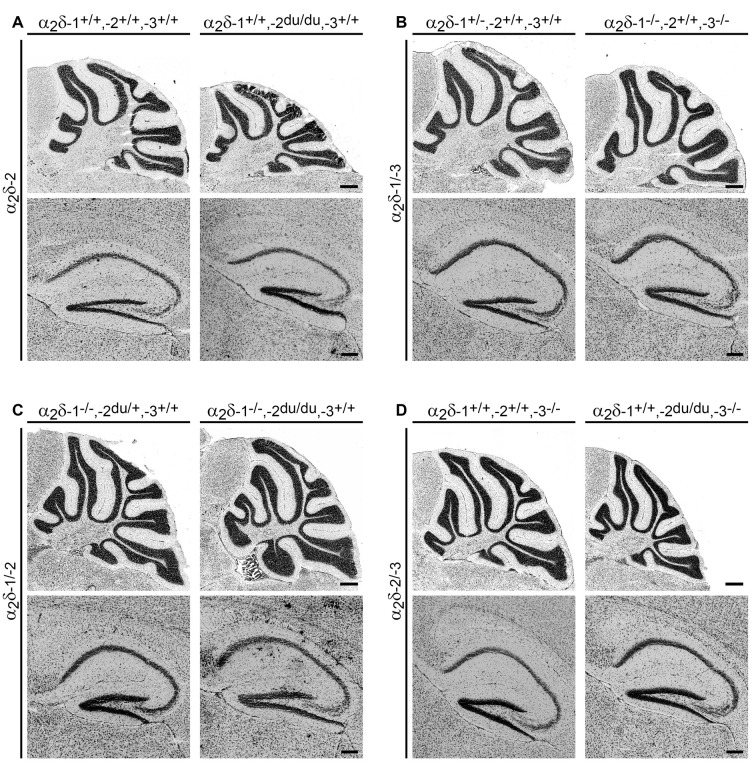
General histologic examination of Nissl-stained brain sections does not reveal major morphological abnormalities. Representative micrographs of Nissl-stained sagittal cryosections obtained from adult (8–13-weeks-old; **A,B**) and juvenile (3–4-weeks-old; **C,D**) mouse brains. The cerebellum and hippocampus of ducky **(A)**, α_2_δ-1/-3 **(B)**, α_2_δ-1/-2 **(C)**, α_2_δ-2/-3 **(D)** double knockout mice showed no overt anatomical defects compared to control mice. Scale bars, 400 μm (Cerebellum), and 200 μm (Hippocampus).

**Figure 4:**
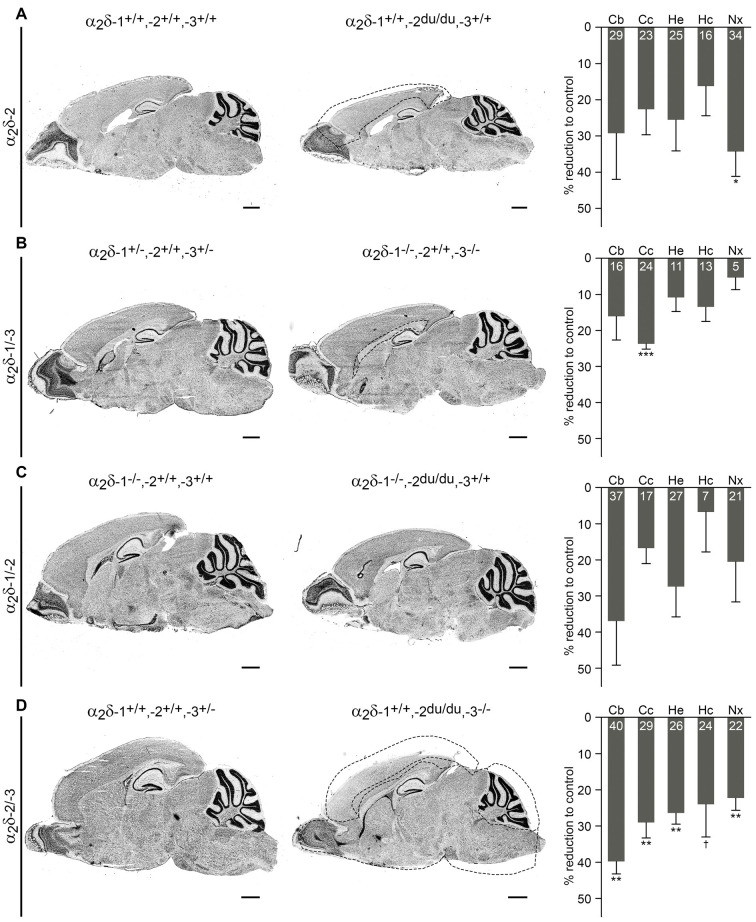
Volumes of distinct brain regions are decreased in adult ducky and α_2_δ double knockout mice. Representative micrographs of Nissl-stained sagittal cryosections obtained from adult (8–13-weeks-old; **A,B**) and juvenile (3–4-week-old; **C,D**) mice. Consecutive slides were used for volume quantification of specific brain regions by applying the Cavalieri principle. Data from three mice per genotype were averaged and bar graphs depict means of respective knockout mice ± SEM, calculated as percentage reduction to corresponding controls (indicated with numbers in bars). Dashed lines in micrographs illustrate significantly decreased brain areas in knockout mice (right picture of each panel) compared to respective controls (left picture of each panel). **(A)** Ducky mice showed a generally reduced volume of all analyzed brain regions, with neocortical size being significantly decreased by 34%. **(B**) While the majority of analyzed structures were only slightly affected in α_2_δ-1/-3 double knockout mice, a specific volume reduction of 24% was found in the corpus callosum. **(C)** Brain regions of α_2_δ-1/-2 double knockout mice were not significantly different from control animals which already lacked α_2_δ-1. However, additional knockout of α_2_δ-2 caused an obvious trend towards reduced volumes of the cerebellum (37%), whole hemisphere (27%), and neocortex (21%). **(D)** α_2_δ-2/-3 double knockout mice showed a drastic volume reduction of all analyzed brain regions ranging from 22 to 40%. The highly significant decrease of the cerebellum, whole hemisphere, and neocortex was similar to the one found in α_2_δ-1/-2 double knockout mice. Furthermore, the size of the corpus callosum was significantly reduced by 29%. Confer Supplementary Table 2 for raw data of individual mice and respective genotypes. *Abbreviations*: Cb, cerebellum, Cc, corpus callosum, He, whole hemisphere, Hc, hippocampus, Nx, neocortex. *Statistics*: unpaired *t*-test with Holm–Sidak correction for multiplicity (for *p*-values see Supplementary Table 2). Symbols in graphs indicate corrected significance levels compared to control: ^†^*p* < 0.06; *p < 0.05; **p < 0.01; ***p < 0.001. Scale bars, 1 mm.

## Results

### Generation of α_2_δ Double Knockout Mice

Loss-of-function models such as knockout or mutant animals allow conclusions on the potential roles of the affected proteins. Of the existing α_2_δ subunit loss-of-function mouse models only the α_2_δ-2 mutant mice, in our case ducky (α_2_δ-2^du/du^), essentially an α_2_δ-2 null mouse (see “Materials and Methods” section), display a severe neurological CNS phenotype and decreased life span (Meier, 1968; Barclay et al., 2001; Brodbeck et al., 2002). Thus, we hypothesized that the neuronal α_2_δ isoforms (α_2_δ-1, α_2_δ-2, and α_2_δ-3) may have partially redundant as well as specific functions. To address this hypothesis, we generated three distinct α_2_δ double knockout mouse models by crossbreeding single knockout (α_2_δ-1 and -3) or mutant (ducky) mice (see below and parental genotypes in Figure 1).

Due to the phenotypes of the distinct single knockout mice—ducky mice are infertile and male α_2_δ-1 knockout mice exhibit a progressive form of diabetes (Snell, 1955; Mastrolia et al., 2017)—successful generation of double knockouts was inherently difficult and accomplished only by employing the following strategies: ducky mice (α_2_δ-2^du/du^) were generated by breeding heterozygous mice (α_2_δ-2^du/+^ × α_2_δ-2^du/+^; Figure 1A). α_2_δ-1/-3 double knockout mice (α_2_δ-1^−/−^, α_2_δ-3^−/−^) were either obtained by inter-crossing double heterozygous animals (α_2_δ-1^+^^/–^, α_2_δ-3^+^^/–^), or mice heterozygous for α_2_δ-1 (α_2_δ-1^+^^/–^) and homozygous knockout for α_2_δ-3 (α_2_δ-3^−/−^; Figure 1B). α_2_δ-1/-2 double knockout/mutant mice (α_2_δ-1^−/−^, α_2_δ-2^du/du^) were generated by cross-breeding double heterozygous animals (α_2_δ-1^+^^/–^, α_2_δ-2^du/+^), or mice heterozygous for α_2_δ-2 (α_2_δ-2^du/+^) and homozygous knockout for α_2_δ-1 (α_2_δ-1^−/−^; Figure 1C). Finally, α_2_δ-2/-3 double knockout/mutant mice (α_2_δ-2^du/du^, α_2_δ-3^−/−^) were obtained by inter-crossing double heterozygous animals (α_2_δ-2^du/+^, α_2_δ-3^+^^/–^), or mice heterozygous for α_2_δ-2 (α_2_δ-2^du/+^) and homozygous knockout for α_2_δ-3 (α_2_δ-3^−/−^; Figure 1D). Most of the different breeding combinations did not yield the respective double knockout mice at the expected Mendelian ratios (see below). Hence, we observed that individual double knockouts were best obtained using the following male and female genotype combinations: α_2_δ-1/-3 double knockout mice by cross-breeding male α_2_δ-1^+/-^, α_2_δ-3^+^^/–^, or α_2_δ-3^−/−^ mice and female α_2_δ-1^+^^/–^, α_2_δ-3^+^^/–^, or α_2_δ-3^−/−^ mice (Figure 1B); α_2_δ-1/-2 double knockout mice by cross-breeding male α_2_δ-1^−/−^, α_2_δ-2^du/+^, and female α_2_δ-1^−/−^, α_2_δ-2^du/+^ mice (Figure 1C); α_2_δ-2/-3 double knockout mice by cross-breeding male α_2_δ-2^du/+^, α_2_δ-3^−/−^, and female α_2_δ-2^du/+^, α_2_δ-3^+^^/–^ mice (Figure 1D). For further analysis, double knockout mice obtained from these breeding combinations were compared with wildtype, (double-) heterozygous, or single knockout littermates.

### Mendelian Ratios are Altered in Neonatal Litters Bred From Different α_2_δ Inter-crosses

We first carried out breeding and offspring analysis in the ducky α_2_δ-2 mouse line and the three double knockout mouse strains. When cross-breeding male and female mice heterozygous for α_2_δ-2 (α_2_δ-2^du/+^ × α_2_δ-2^du/+^; Figure 1A) we observed that the detected genotypes in P0–1 litters were close to expected theoretical values for wildtype, heterozygous and ducky mice.

However, the genotypic distribution of neonatal litters in the double heterozygous breeding combinations did not conform to Mendel’s law: the frequency of heterozygous pups was generally increased by 15–36% compared to expected percentages. In striking contrast, the prevalence of α_2_δ double knockout mice was ~30% (α_2_δ-1/-3; Figure 1B, left), 100% (α_2_δ-1/-2; Figure 1C, left), and 80% (α_2_δ-2/-3; Figure 1D, left) less than theoretically expected at birth, and even the number of single knockout pups was significantly reduced. The ratio of observed and predicted single knockout mice varied in the distinct α_2_δ cross-breedings: while the numbers of born α_2_δ-2 mutant pups were reduced by 40% (α_2_δ-1/-2; Figure 1C, left) and 100% (α_2_δ-2/-3; Figure 1D, left), α_2_δ-1 or α_2_δ-3 single knockouts were close to or slightly above expected values in the respective mouse lines. In α_2_δ-1/-3 inter-crosses (Figure 1B, left) the amount of born α_2_δ-1 single knockout mice was 70% less than predicted, whereas 80% more α_2_δ-3 single knockout mice were born.

Using male or female mice of different genotype combinations revealed further effects on genotype frequency in neonatal litters: cross-breeding male α_2_δ-1^+^^/–^, α_2_δ-3^−/−^, and female α_2_δ-1^+^^/–^, α_2_δ-3^+^^/–^ mice more strongly affected mendelian distribution compared with double heterozygous breedings, as the number of born single and double knockout pups was decreased by 32% and 68%, respectively (Figure 1B, right). Of note, the chance to obtain α_2_δ-1/-2 double knockout pups was strikingly low in all α_2_δ-1/-2 breeding combinations (Figure 1C, 73% or 100% less than expected). The observed genotypes were close to expected theoretical values by inter-crossing male and female α_2_δ-2^du/+^, α_2_δ-3^−/−^ mice (Figure 1D, right) and thus provided a relatively good chance to obtain α_2_δ-2/-3 double knockout mice. Taken together, genotypes of parents were a critical factor for the birth/survival of single and double knockout offspring. This is best visualized by the total number of analyzed offspring and litters (Table 1): While data from heterozygous α_2_δ-2 matings were collected within 2 years, double knockout mouse lines required a period of 4–5 years to collect enough data enabling statistical analysis. Taken together, our results suggest embryonic or neonatal lethality of distinct α_2_δ single and double knockout mice, therefore indicating that α_2_δ subunits are essential for survival.

**Table 1:** Mean litter sizes obtained from distinct α_2_Δ mutant or knockout breeding combinations.

Breeding pair combination ♂ X ♀ (number of pairs)	Litter size (mean ± SD)	Total number of offspring	Total number of litters	Period of data collection (years)
α_2_Δ-2^du/+^ X α_2_δ-2^du/+^ (11)	8.3 ± 2.1	178	21	2
α_2_Δ−1^+/–^, α_2_δ-3^+/–^ X α_2_Δ−1^+/–^, α_2_Δ-3^+/–^ (8)	7.6 ± 2.7	159	21	5
α_2_Δ−1^+/–^, α_2_δ-3^−/–^ X α_2_Δ−1^+/–^, α_2_δ-3^+/–^ (2)	7.8 ± 2.5	47	6	2.5
α_2_Δ−1^+/–^, α_2_δ-2^du/+^ X α_2_Δ−1^+/–^, α_2_Δ-2^du/+^ (6)	6.3 ± 3.3	107	17	2
α_2_Δ-1^−/–^, α_2_Δ-2^du/+^ X α_2_Δ-1^+/–^, α_2_Δ-2^du/+^ (3)	6.0 ± 2.1	30	5	4
α_2_Δ-1^−/–^, α_2_Δ-2^du/+^ X α_2_Δ-1^−/–^, α_2_Δ-2^du/+^ (9)	4.5 ± 1.7	59	13	4
α_2_Δ-2^du/+^, α_2_Δ-3^+/–^ X α_2_Δ-2^du/+^, α_2_Δ-3^+/–^ (4)	7.8 ± 2.7	94	12	2
α_2_Δ-2^du/+^, α_2_Δ-3^−/–^ X α_2_Δ-2^du/+^, α_2_Δ-3^+/–^ (7)	7.2 ± 2.5	72	10	4
α_2_Δ-2^du/+^, α_2_Δ-3^−/–^ X α_2_Δ-2^du/+^, α_2_Δ-3^−/–^ (10)	7.9 ± 1.0	103	13	4

### Breeding Pairs of Distinct α_2_δ-2/-3 Inter-crosses Display Skin Lesions Associated With Over-Grooming

Since genotypes of parents appeared to be a critical factor for the survival of single and double knockout offspring, we more thoroughly monitored breeding pairs. Besides the diabetic phenotype of male α_2_δ-1 knockout animals (Mastrolia et al., 2017), no overt physiological or behavioral abnormalities have been observed in α_2_δ-2, α_2_δ-1/-3, and α_2_δ-1/-2 mating combinations. In contrast, already α_2_δ-2/-3 double heterozygous breeding pairs displayed signs of stress and anxiety-like behavior typically associated with neuropsychiatric disorders (Kalueff et al., 2016; Landmann et al., 2018a) comprising head trembling, increased grooming, and abnormal activity upon handling. Excessive grooming appeared enhanced in double heterozygous and even more in heterozygous/knockout matings, as we observed high incidences of skin lesions (Table 2, double heterozygous breeding: no overt lesions in six breeding cages; α_2_δ-2^du/+^, α_2_δ-3^−/−^ × α_2_δ-2^du/+^, α_2_δ-3^+^^/–^: lesions observed in 28% of 14 breeding cages). Of note, in α_2_δ-2^du/+^, α_2_δ-3^−/−^ × α_2_δ-2^du/+^, α_2_δ-3^+^^/–^ matings lesions were observed in 83% of 24 breeding cages which correlated with extraordinarily high infant mortality at P0–1, often resulting in the loss of complete litters. Thus, breedings were later switched to include BALB/c foster mothers (see “Materials and Methods” section). Similar incidences (loss of litters) have also been detected in α_2_δ-1/-2 matings, although here no excessive grooming behavior was observed.

**Table 2:** α_2_δ-2/-3 breeding pairs display abnormal grooming behavior and increased pup mortality.

Genotype female	Genotype male	Skin lesions in breeders	^a^Number of litters with infant mortality	Number of analyzed breeding pairs
α_2_δ-2^du/+^, α_2_δ-3^+^^/–^	α_2_δ-2^du/+^, α_2_δ-3^+^^/–^	♀ 0% ♂ 0%	6 of 24	6
α_2_δ-2^du/+^, α_2_δ-3^+^^/–^	α_2_δ-2^du/+^, α_2_δ-3^−/–^	♀ 0% ♂ 36%	3 of 8	11
α_2_δ-2^du/+^, α_2_δ-3^−/–^	α_2_δ-2^du/+^, α_2_δ-3^+^^/–^	♀ 0% ♂ 0%	4 of 24	3
α_2_δ-2^du/+^, α_2_δ-3^−/–^	α_2_δ-2^du/+^, α_2_δ-3^−/–^	♀ 67% ♂ 50%	23 of 31	24

*^a^Inclusion criteria: ≥25% of the pups from one litter found dead*.

Lesions were usually first noticed within the first month after placing mating mice together and appeared as a patch of hairless skin at specific body regions including the head (neck, eyes, snout, and ears), back, belly, and genitals. Thorough monitoring of mice to ensure the timely use of humane endpoints revealed that the lesions quickly progressed into subcutaneous wounds encompassing larger parts. Because males and females were housed together during the entire breeding procedure we could not definitely determine whether lesions were caused by self- grooming, allogrooming, or could be the result of aggressive encounters. However, partners were not observed behaving aggressively against each other, and individuals were often seen engaged in scratching wounds themselves. Importantly, hardly any occurrences were observed in mice maintained in stock cages of same-sex and same-age littermates. Moreover, lesions were neither noticed in other mouse lines maintained in a C57BL/6N background (α_2_δ-2 and α_2_δ-3 single breedings) nor in the ones kept in a mixed 129J × C57BL/6N background (α_2_δ-1/-3 and α_2_δ-1/-2 double breedings). Thus, our findings rather exclude the possibility of background related symptoms or diseases and point towards abnormal behavior caused by the gradual decrease in the total amount of α_2_δ-2 and particularly α_2_δ-3.

### General Descriptions of Phenotypes of α_2_δ-2 Null (Ducky) Mice and α_2_δ Double Knockout Mice

We subsequently conducted a qualitative phenotypic evaluation looking for gross abnormalities in born ducky and double knockout offspring (see “Materials and Methods” section).

Similar to previously published reports, the α_2_δ-2 mutant mice analyzed in this study displayed the ducky phenotype, described by an ataxic, wide-based gait (Snell, 1955) as well as symptoms typical for epileptic seizures including loss of balance, falling to the side, and behavioral immobility (Van Erum et al., 2019). However, while other studies stated that the majority of ducky mice hardly lived beyond 39 days (Meier, 1968; Barclay et al., 2001), we observed in several animals a highly extended life span (>12 months; Schoepf et al., 2019). The discrepancy in survival might be explained by the use of different background strains or the above-mentioned husbandry strategies including easily reachable food and water (see “Materials and Methods” section). Notably, constant monitoring revealed that adult mice exhibited to some extent normal activities such as climbing on the housing material and huddling behavior, all signs of general well-being.

Similar to ducky mice, α_2_δ-1/-2 and α_2_δ-2/-3 double knockout mice also showed abnormal behavioral phenotypes typically occurring during apparent epileptic episodes (see paragraph above). However, in contrast to ducky animals, α_2_δ-1/-2 and α_2_δ-2/-3 double knockout mice displayed a more severe disease progression peaking around weaning age, which often required the application of humane endpoints (see “Materials and Methods” section). Their generally affected condition was best evidenced by poor fur coat condition, low body weight (Figure 2), hypo-reactivity to handling, and highly reduced survival chance during the first postnatal weeks (Schoepf et al., 2019). We did therefore not pursue quantitative behavioral tests of sensory or motor abilities as these mice would have been unable to perform such tasks. Interestingly, while no motor phenotype has been so far detected in α_2_δ-1 and α_2_δ-3 single knockout animals, α_2_δ-1/-3 double knockout mice showed locomotion difficulties comprising imbalance and abnormal posture while walking. However, gait appeared different compared to the previously described waddling gait of ducky mice, which was also evident in α_2_δ-1/-2 and α_2_δ-2/-3 knockout mice. When further examined, α_2_δ-2 null mice displayed a frog-like position of the hind limbs as described in Meier (1968), whereas α_2_δ-1/-3 double knockout animals exhibited a hopping gait lifting one of the hindlimbs. Notably, quantitative gait assessment using the CatWalk gait analysis system for fluorescent detection of footprint patterns already failed in α_2_δ-1/-3 double knockout mice because of their highly reduced body weight (Figure 2).

### Loss of Distinct α_2_δ Subunits Causes Impaired Development and Neurological Disease

To further characterize how the loss of distinct α_2_δ subunits affects postnatal development and neurological disease we next assessed body and brain weights of juvenile (3-4-week-old; α_2_δ-2/-3 double knockout and ducky) or adult (8–13-week-old; α_2_δ-1/-3 double knockout and ducky) male mice. Moreover, brain/body ratios were calculated to investigate whether an eventual decrement of brain size was associated with developmental impairment. Since delivery of α_2_δ-1/-2 double knockout pups was rare (Figure 1C) they could not be included in this analysis. We observed that in all three analyzed mouse models juvenile mice showed a highly decreased bodyweight (ducky: 41%; α_2_δ-1/-3: 61%; and α_2_δ-2/-3: 68% reduction compared to control) together with a moderately reduced brain size (ducky: 10%; α_2_δ-1/-3: 22%; and α_2_δ-2/-3: 28% reduction to control) resulting in 1.5-fold (Figure 2A; ducky), 2-fold (Figure 2B; α_2_δ-1/-3); or 2.2-fold (Figures 2C,E; α_2_δ-2/-3), enhanced brain/body ratios compared to individual controls. Interestingly, differences in brain/body ratios were no longer apparent in adult α_2_δ-1/-3 double knockout mice (Figure 2B). In contrast, adult ducky mice (Figure 2A; 2.4-fold) showed even higher brain/body ratios due to a relatively mild increase in body size during adolescence and unaltered brain weight (body: 61%; brain: 17% reduction to control). Moreover, comparing juvenile α_2_δ-2 and α_2_δ-3 single knockout mice with α_2_δ-2/-3 double knockout littermates further corroborated that loss of distinct α_2_δ subunits differentially affected brain and body size: while α_2_δ-3 knockout mice had normal brain/body ratios compared to controls (Figure 2C, blue squares), brain/body ratios were strongly increased in α_2_δ-2 null mice (Figure 2C, green squares, 1.4-fold) and even more dramatically in α_2_δ-2/-3 double knockout mice (Figure 2C, magenta squares, 2.2-fold). Together, these data are in accordance with the above-mentioned severe neurological phenotypes associated with the lack of α_2_δ-2. These phenotypes were even stronger if α_2_δ-3 or, most likely, α_2_δ-1 were additionally missing in double knockout mice. Furthermore, our findings suggest that α_2_δ-2 and α_2_δ-3 differentially affect postnatal growth and brain development.

### Volumes of Distinct Brain Regions Are Decreased in Ducky and Double Knockout Mice

Interestingly, we observed that the brain weight of ducky and double knockout mice was moderately to highly reduced compared to control littermates (Figure 2). Given the concomitant decrease in body weight, we reasoned that the decrement in brain size could be caused by a general developmental delay. If this applies, we assumed that distinct brain regions should be rather uniformly affected by the loss of α_2_δ subunits. However, taking the distinct phenotypes of the individual single and double knockout mice into account, α_2_δ subunits may also specifically influence the development of brain regions. In this scenario, the loss of α_2_δ subunits could lead to decreased volumes of discrete brain regions. Thus, we next examined overall brain architecture in juvenile (α_2_δ-1/-2 and α_2_δ-2/-3 double knockout) or adult (α_2_δ-1/-3 double knockout) male mice. To our knowledge ducky mice of over 56-days of age have not been histologically examined thus far (Meier, 1968; Brodbeck et al., 2002) and were therefore included in our analysis.

To address potential α_2_δ knockout effects on brain structure, consecutive Nissl-stained sagittal cryosections from mutant brains obtained from one hemisphere were compared with control littermates. Moreover, volumes of distinct brain regions of interest, including the cerebellum, corpus callosum, hippocampus, neocortex, and whole hemisphere were calculated by applying the Cavalieri principle (see “Materials and Methods” section for selection criteria). While we found no overt anatomical abnormalities in cerebelli or hippocampi of all α_2_δ mutant and ducky mice (Figure 3), the volumetric analysis revealed size differences of specific brain regions (Figure 4 and Supplementary Table 2): whole hemisphere volume of α_2_δ-1/-3 double knockout mice was slightly reduced by 11% (Figure 4B), while brain size of ducky (Figure 4A), α_2_δ-1/-2 (Figure 4C) and α_2_δ-2/-3 (Figure 4D) double knockout mice was strongly decreased (25%, 27%, and 26%, respectively). Furthermore, the size of the cerebellum was markedly affected in α_2_δ-2/-3 (40%) and α_2_δ-1/-2 (37%) double knockout mice and to a lesser extent in ducky (29%) and α_2_δ-1/-3 mice (16%). Interestingly, cortical volume was decreased in all three mouse strains lacking the α_2_δ-2 isoform (ducky: 34%, α_2_δ-1/-2: 21% and α_2_δ-2/-3: 22%), while the corpus callosum was significantly reduced whenever α_2_δ-3 was absent (α_2_δ-1/-3: 23%, α_2_δ-2/-3: 29%). Of note, albeit some alterations were not significantly different to controls, which might be linked to low n-numbers caused by inherently difficult breeding strategies and phenotypes, they were consistently detected in all mutant animals analyzed (Supplementary Table 2: cerebellum in α_2_δ-1/-2; corpus callosum in ducky and α_2_δ-1/-2; hippocampus in α_2_δ-1/-3; neocortex in α_2_δ-1/-2; whole hemisphere in ducky, α_2_δ-1/-3 and α_2_δ-1/-2). Taken together, our data display concurrent effects on specific brain regions caused by deletion of distinct α_2_δ subunits: sizes of cerebellum and cortex were highly reduced when α_2_δ-2 was absent, and a lack of α_2_δ-3 specifically elicited a volume decrease in the corpus callosum. Therefore, our findings argue that the reduction in brain size is not just caused by a general effect on overall development.

### Adult Ducky Mice Display a Reduced Thickness of Cortical Layers and Increased Cell Densities

A potentially underlying cause for a reduction in the volume of distinct brain regions in α_2_δ mutant mice could be on the one hand loss of cells or on the other hand a paucity of neuropil, which includes neuronal and glial cell processes, dendritic spines, and synaptic contacts (Dudanova et al., 2007). The neocortex provides an intriguing brain region to further address these possibilities and thus shed light onto the role of α_2_δ subunits in the normal and diseased brain in several aspects: first, we found that the cortical volume was decreased in all three mouse models lacking the α_2_δ-2 isoform (Figure 4, see ducky, α_2_δ-1/-2 and α_2_δ-2/-3), which has not been described in the original reports of young ducky mice (Meier, 1968). Second, abnormal structure and function of the cerebral cortex have been linked to neurodevelopmental and neurological disease, including autism spectrum disorders (Chen et al., 2015; Fenlon et al., 2015), motor dysfunction (Hong and Mah, 2015), and epilepsy (Toba and Hirotsune, 2012). Importantly, these phenotypes have been associated with aberrant α_2_δ expression both in mice and humans (reviewed in Geisler et al., 2015). Third, the cerebral cortex represents a six-layered structure that is generated during corticogenesis in a highly regulated manner: earlier born neurons reside in deeper layers, and later-born neurons migrate to superficial layers (Shao et al., 2017). Consequently, an effect of α_2_δ subunit mutations should also be examined during development.

**Figure 5:**
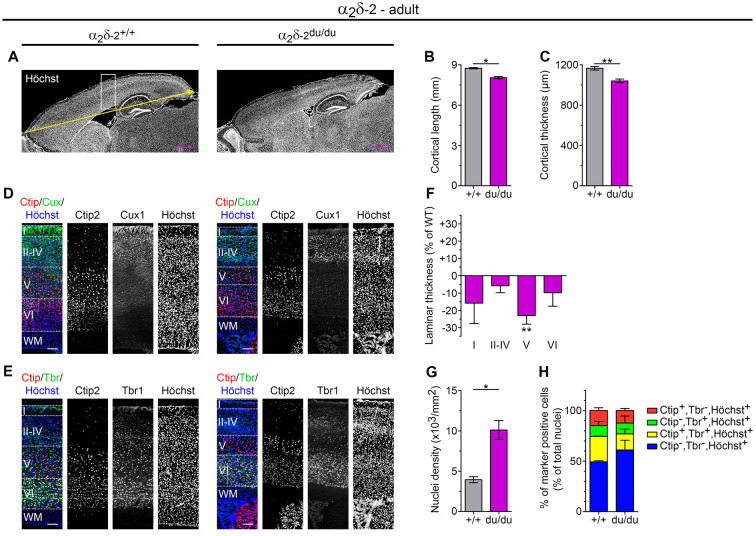
Adult ducky mice display a reduced thickness of cortical layers and increased cell densities. **(A–C)** Representative micrographs of mid-sagittal cryosections obtained from adult (8–13-weeks-old) wildtype and α_2_δ-2 mutant (ducky) mouse brains **(A)**. Slides were counterstained with the nuclear marker Höchst to analyze anterior-posterior length (yellow arrow and quantification in panel **(B)** and thickness **(C)** of the somatosensory cortex, which were both significantly decreased in ducky mice. Two **(B)** and three **(C)** mice per genotype were analyzed and bar graphs depict means of mice ± SEM. *Statistics*: unpaired *t*-test: **(B)** *t*_(2)_:6.9, *p* = 0.02; **(C)** *t*_(4)_:4.8, *p* = 0.009. Representative micrographs of triple immunofluorescence labelings of consecutive sagittal sections with layer-specific markers Ctip2 (red; layer V), Cux1 (green; layer II–IV) or Tbr1 (green; layer VI) and Höchst (blue) at the level of somatosensory cortex (see the boxed region in **A**), showing that cortical lamination is preserved in adult ducky mice **(D,E)**. Quantification of the laminar thickness **(F)**, total cell density **(G)**, and percentage of marker positive neurons of total cells **(H)**. The thickness of individual layers was reduced in ducky mice when compared to wild-type controls, the most affected being layer V (**F**, 23%). This effect was accompanied by a 2.5-fold increase in nuclear cell density **(G)** while the proportion of cells expressing individual markers remained unaffected **(H)**. Two mice per genotype were analyzed and bar graphs depict means of mice ± SEM.* Statistics*: **(F)** Two-way RM ANOVA with Holm–Sidak *posthoc* analysis: genotype: *F*_(1,6)_ = 17.9, *p* = 0.05, layer: *F*_(3,6)_ = 150.5, *p* < 0.001, genotype x layer: *F*_(3,6)_ = 1.4, *p* = 0.34, *posthoc*: **p < 0.001 between ducky and wildtype within layer 5; **(G)** unpaired *t*-test: *t*_2_:5.0, *p* = 0.038; **(H)** Two-way RM ANOVA with Holm–Sidak *posthoc* analysis: genotype: *F*_(1,6)_ = 0.6, *p* = 0.5, marker: *F*_(3,6)_ = 26.3, *p* < 0.001, genotype x marker: *F*_(3,6)_ = 1.2, *p* = 0.39. Symbols in graphs indicate significance levels: *p < 0.05; **p < 0.01. Scale bars, 1 mm (**A,B**) and 100 μm (**D,E**).

Therefore, we next aimed to discern the relative contribution of cell densities to the cortical volume, which represents a reflection of spacing between cell somas (Chen et al., 2015). To this end, cortical cytoarchitecture of adult ducky and wild-type mice was analyzed measuring a battery of distinct parameters (Figure 5). Using sagittal cryosections counterstained with the nuclear marker Höchst (Figure 5A) we found that both anteroposterior length (Figure 5B) and thickness of the somatosensory cortex (Figure 5C) were significantly reduced by 10% compared to wildtype control littermates. These findings further validate the volume decrease observed on Nissl-stained cryosections (Figure 4A). Because total cortical volume was reduced by 34% in α_2_δ-2 mutant mice (Figure 4A), these data likely suggest that cortical length is decreased to a similar extent along the rostrocaudal (Figure 5B), ventrodorsal (Figure 5C), and possibly also the mediolateral axis, therefore displaying a rather general effect on cortical expansion. Importantly, measuring nuclear densities along the entire ventrodorsal axis showed that the effect of reduced thickness was accompanied by a 2.5-fold increase in cell density (Figure 5G, number of all Höchst positive cells/mm^2^ encompassing layer I to layer VI). Together, these results point towards a more compacted cortex in adult ducky mice compared to wild-type control littermates.

To evaluate whether the reduction in cortical thickness was linked to an altered layer composition we subsequently examined cortical lamination using well-established markers for upper-layer (Cux1: layer II–IV; Figure 5D) and deep-layer neurons (Ctip2: layer V; Tbr1: layer VI; Figure 5E; Shao et al., 2017). Triple immunofluorescence labeling of consecutive sagittal cryosections with Ctip2 (red channel) and either Cux1 or Tbr1 (green channel), as well as Höchst (blue channel), revealed that cortical lamination appeared preserved in adult ducky mice (Figures 5D,E, right panel). However, laminar thickness at the level of the somatosensory cortex was generally reduced in ducky mice, albeit the most severely and consistently affected being layer V (23% reduction to control; Figure 5F). Because the proportion of marker positive cells relative to the number of Höchst^+^ cells was not apparently altered (Figure 5H and Supplementary Table 3) our data imply that cortical thinning in adult ducky mice is primarily caused by decreased spacing, possibly reflecting a reduction of dendritic arborization, axonal contacts, and synapses.

### Cortical Thinning and Compaction Manifests During Postnatal Development in Ducky Mice

**Figure 6:**
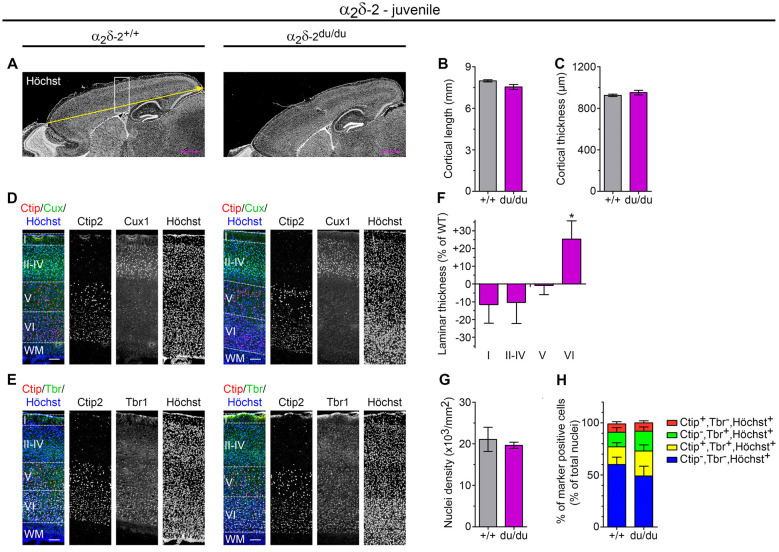
Juvenile ducky mice display altered cortical cytoarchitecture. **(A–C)** Representative micrographs of mid-sagittal cryosections obtained from juvenile (3-weeks-old) wildtype and α_2_δ-2 mutant (ducky) mouse brains **(A)**. Slides were counterstained with the nuclear marker Höchst to analyze anterior-posterior length (yellow arrow and quantification in panel **B**) and thickness **(C)** of the somatosensory cortex. Both cortex length and cortical thickness of ducky mice did not significantly differ compared to wildtypes. Four mice per genotype were analyzed and bar graphs depict means of mice ± SEM. *Statistics*: unpaired *t*-test: **(B)** *t*_6_: 2.3, *p* = 0.06; **(C)** *t*_(6)_: 1.1, *p* = 0.33. Representative micrographs of triple immunofluorescence labelings of consecutive sagittal sections with layer-specific markers Ctip2 (red; layer V), Cux1 (green; layer II–IV) or Tbr1 (green; layer VI) and Höchst (blue) at the level of somatosensory cortex (see the boxed region in **A**), indicating lamination is preserved in juvenile ducky mice **(D,E)**. However, further quantitative analysis of laminar thickness **(F)**, total cell density **(G)**, and percentage of marker positive neurons of total cells **(H)** revealed a significant increase of layer VI in juvenile ducky mice (**F**, 25%) without apparent effects on total cell density **(G)** or proportion of marker positive cells **(H)**. Four mice per genotype were analyzed and bar graphs depict means of mice ± SEM.* Statistics*: **(F)** two-way RM ANOVA with Holm–Sidak *posthoc* analysis: genotype: *F*_(1,18)_ = 0.17, *p* = 0.7, layer: *F*_(3,18)_ = 124.7, *p* < 0.001, genotype × layer: *F*_(3,18)_ = 2.5, *p* = 0.095, *posthoc*: *p < 0.05 between ducky and wildtype within layer 6; **(G)** unpaired *t*-test: *t*_(6)_: 5.0, *p* = 0.65; **(H)** two-way RM ANOVA with Holm–Sidak *post hoc* analysis: genotype: *F*_(1,18)_ = 0.83, *p* = 0.4, marker: *F*_(3,18)_ = 21.6, *p* < 0.001, genotype × marker: *F*_(3,18)_ = 0.87, *p* = 0.48. Symbols in graphs indicate significance levels: *p < 0.05. Scale bars, 1 mm **(A,B)** and 100 μm **(D,E)**.

To assess whether cortex size was already affected at earlier postnatal stages which would point towards a growth deficit, we next examined juvenile ducky mice and compared them to wildtype control littermates (3–4-week-old). Surprisingly, we found that the anteroposterior length was only slightly reduced by 6% (Figures 6A,B, *p* = 0.06) and cortical thickness was unaltered compared to wildtypes (Figure 6C, *p* = 0.33). Together, these findings suggest that, in contrast to adult ducky mice, total cortical expansion was still unaffected in juvenile mice. Since also total nuclear cell density was comparable to wildtype control (Figure 6G, number of all Höchst positive cells/mm^2^ encompassing layer I to layer VI) our data further implicate that cortical thinning and compaction did not yet manifest in juvenile mice. Interestingly, however, immunofluorescence labeling of consecutive sagittal cryosections with layer-specific markers for upper-layer (Cux1: layer II–IV; Figure 6D) and deep-layer neurons (Ctip2: layer V; Tbr1: layer VI; Figure 6E) revealed differences in lamination: while layers I and II–IV showed a trend towards reduced thicknesses by 10%, layer VI displayed a concomitant increase of 25% (Figure 6E, right panel, and Figure 6F). Similar to adult ducky mice, we next assessed whether the increase in layer VI was linked to a relative increase in deep-layer neurons by calculating the fraction of marker positive cells relative to the number of Höchst^+^ cells (Figure 6H and Supplementary Table 3). Since the proportion was not altered, our findings indicate that increased laminar thickness might be a result of augmented spacing in juvenile α_2_δ-2 mutant mice.

Altogether, the above-described divergent observations in cortices of adult and juvenile ducky mice possibly reflect a reduction of spacing in adult mice which manifests after 3–4 weeks of age. Moreover, our results suggest that loss of neuronal α_2_δ subunits causes a paucity of neuropil, which includes neuronal and glial cell processes, spines, and synaptic contacts. Thus, the reduction of cortex volume cannot be solely explained by developmental retardation, further implicating the role of neuronal α_2_δ subunits in the stabilization of axonal and dendritic arborization.

## Discussion

This is the first study providing a general assessment of behavioral phenotypes and systemic analysis of different brain regions of three newly established α_2_δ double knockout mouse models (α_2_δ-1/-3, α_2_δ-1/-2, and α_2_δ-2/-3) and adult α_2_δ-2 mutant ducky mice. The findings described here provide evidence for general essential, but also individual roles of α_2_δ subunit isoforms in regulating survival, behavior, postnatal development, and the size of distinct brain regions. Importantly, we demonstrate for the first time that neurological disease phenotypes and the level of their severity critically depend on the type and quantity of α_2_δ isoforms.

### Role of α_2_δ Isoforms in Premature Survival

Our offspring analysis on neonatal litters (P0–1) obtained by crossbreeding distinct α_2_δ-1, α_2_δ-2, and α_2_δ-3 genotype combinations revealed that genotypic distribution did not conform to Mendel’s law, thus suggesting embryonic or neonatal lethality of α_2_δ-1/-3, α_2_δ-1/-2, and α_2_δ-2/-3 double knockout mice. Together with the fact that α_2_δ single knockout or mutant mice are born at expected Mendelian ratios in litters obtained from heterozygous breeding pairs, this finding may indicate a significant degree of α_2_δ subunit redundancy in premature survival and/or early development (α_2_δ-1: Mastrolia et al., 2017; α_2_δ-2: Figure 1A, present study; α_2_δ-3: Neely et al., 2010; Landmann et al., 2018a, b). One intriguing observation presented here certainly is that not only the number of α_2_δ double knockout offspring but already the amount of single knockout pups was significantly reduced in double heterozygous breedings of all α_2_δ inter-crosses (Figures 1B–D, left graphs). This surprising discrepancy between single heterozygous and double heterozygous matings might suggest that even single knockouts display a decreased chance of survival if obtained together with a high number of heterozygous or wildtype siblings. Such premature mortality in offspring could result either from physiological and/or behavioral deficits in mutant parenting animals, pups themselves, or a combination of both.

In the first scenario, behaviorally affected female mice might display reduced parental care-taking abilities or even conduct infanticide (Kuroda and Tsuneoka, 2013), which is supported by the correlation of excessive grooming behavior with extraordinarily high infant mortality. Along these lines, a previous study found abnormal occurrences of infant mortality using α_2_δ-3 knockout mice as breeders, likely associated with reduced maternal care-taking due to deficits in vocalization and hearing (Landmann et al., 2018a). While we did not detect any obvious occurrences in our α_2_δ-3 single knockout mouse line, we specifically observed infant mortality in α_2_δ-1/-2 and α_2_δ-2/-3 matings, which often resulted in the loss of complete litters at P0–1. Therefore, we occasionally added BALB/c foster mothers to pregnant females, which helped to increase the premature survival chance of α_2_δ mutant offspring. Alternatively, given that α_2_δ-2 expression is also detected in the placenta (Klugbauer et al., 1999) and α_2_δ-3 is discussed as a tumor suppressor for breast cancer (Palmieri et al., 2012), deficits in nutrient supply during pregnancy or lactation might be another conceivable cause for the observed premature mortality (Watkin et al., 2008). As our husbandry strategies were adapted to facilitate analysis of juvenile and adult mice future studies should focus on more detailed behavioral investigations of paternal care-taking abilities, including video analysis of feeding behavior with and without the use of foster mothers.

Alternatively, mutant progeny may also suffer from organ dysfunction or so far unidentified defects in embryonic or early postnatal development, which could affect nutrient supply or the pup’s ability to compete with healthy siblings (Kuroda and Tsuneoka, 2013). This notion might be supported by α_2_δ isoform-specific effects we observed on premature survival (Figures 1B–D, left graphs): Mendelian ratios were exclusively reduced in α_2_δ-2 mutant ducky mice obtained from α_2_δ-1/-2 (Figure 1C) or α_2_δ-2/-3 (Figure 1D) double heterozygous breedings, while α_2_δ-1 and α_2_δ-3 single knockouts were born at expected probabilities. Similarly, α_2_δ-1 single knockout mice were detected at a significantly lower frequency than α_2_δ-3 single knockouts obtained from α_2_δ-1/-3 double heterozygous breedings (Figure 1B). These data implicate that α_2_δ-1 and α_2_δ-2 can partially compensate for the loss of α_2_δ-3 in α_2_δ-3 single knockout mice.

At present, our findings indicate a combination of the above-provided explanations for premature death. To ultimately determine the cause of infant mortality in α_2_δ single and double knockout mice in α_2_δ inter-crosses, the challenge of future investigations will be to elucidate the still incompletely understood α_2_δ isoform expression levels in distinct tissues during development. Currently, comparative analysis of α_2_δ protein expression levels is prevented by the limited quality of available isoform-specific antibodies. For example, antibodies against α_2_δ-1 are not suitable for detecting native proteins (Muller et al., 2010) and antibodies against α_2_δ-3 do not allow protein detection at the cellular and sub-cellular level (Stephani et al., 2019).

### Loss of α_2_δ Isoforms Differentially Affects Neurological Disease

We observed that α_2_δ-2 mutant, α_2_δ-1/-2, and α_2_δ-2/-3 double knockout mice displayed the typical ducky phenotype described by an ataxic, wide-based gait and epileptic seizures. However, α_2_δ-1/-2 and α_2_δ-2/-3 double knockout mice showed a more severe disease progression than ducky individuals, which was best visualized by their highly reduced body weights (Figure 2C) and increased mortality during the first month after birth (Schoepf et al., 2019). In contrast, the α_2_δ-1 and α_2_δ-3 single knockout mouse strains used to generate α_2_δ-1/-3 double knockout mice displayed relatively mild neurological phenotypes (Landmann et al., 2018a, b; Zhou et al., 2018). Moreover, no effects on survival (α_2_δ-3), or less severely affected life expectancy (α_2_δ-1) have been demonstrated so far, whereas α_2_δ-1/-3 double knockout mice showed a decreased mean survival comparable to that of ducky mice (3 months; Schoepf et al., 2019). At a first glance, these data indicate redundant functions for α_2_δ isoforms in postnatal survival and development. This notion might be supported by their partly overlapping and relatively ubiquitous distribution in distinct adult organs: α_2_δ-1 and α_2_δ-2 are expressed in the heart, skeletal muscle, and pancreas (Ellis et al., 1988; Klugbauer et al., 1999; Gao et al., 2000; Mastrolia et al., 2017), and α_2_δ-1, α_2_δ-2, and α_2_δ-3 are detected in various regions of the brain (Cole et al., 2005; Schlick et al., 2010; Geisler et al., 2019). Therefore, simultaneous loss of two α_2_δ subunit isoforms might cause developmental defects or physiological dysfunction, which would be normally compensated by remaining isoforms in single knockout mice.

Alternatively, the reduced life spans of α_2_δ-1/-3, α_2_δ-1/-2, and α_2_δ-2/-3 double knockout mice could be related to a compound effect which is triggered by the combination of two α_2_δ isoforms deleted. This idea might be underpinned by the partially tissue-specific expression patterns and distinct disease phenotypes of individual single knockout mice. For instance, deletion of α_2_δ-1 impairs cortical excitatory synaptogenesis (Risher et al., 2018), cardiac function (Fuller-Bicer et al., 2009), and insulin release (Mastrolia et al., 2017) without apparently altering protein (Fuller-Bicer et al., 2009) or mRNA (Mastrolia et al., 2017) expression levels of other isoforms. These reports indicate that α_2_δ-1 either exerts specific functions or that low amounts of the remaining isoforms likely do not suffice to compensate for the loss of α_2_δ-1. Along those lines, the severe consequences of α_2_δ-2 mutations in animal models (Barclay et al., 2001; Brill et al., 2004; Ivanov et al., 2004; Donato et al., 2006) are likely caused by its predominant expression in the cerebellum (Schlick et al., 2010). Notably, α_2_δ-3 knockout mice display overexpression of Ca_V_2 calcium channel subtypes in the brain, possibly resulting in enhanced neuronal excitability (Landmann et al., 2018b). Therefore, the increased postnatal lethality and severe neurological disease of α_2_δ-1/-3, α_2_δ-1/-2, and α_2_δ-2/-3 double knockout animals might be related to a synthetic lethal effect, as suggested previously for the synaptic cell adhesion molecules neuroligins (Varoqueaux et al., 2006). Here, α_2_δ isoform-specific mutant phenotypes in distinct tissues possibly accumulate, leading to dysfunction of organs or pivotal brain networks.

Several principal observations argue for distinct disease mechanisms in the individual mouse models: first, ducky, α_2_δ-1/-2, and α_2_δ-2/-3 double knockout mice showed apparent epileptic episodes, while similar seizures during handling were never observed in α_2_δ-1/-3 double knockout mice. Second, even though α_2_δ-1/-3 double knockout mice displayed abnormal locomotion, gait appeared different compared to the typical waddling gait of ducky, α_2_δ-1/-2, and α_2_δ-2/-3 knockout mice. Third, breeding pairs of distinct α_2_δ-2/-3 inter-crosses exhibited abnormal behavior including self-injurious repetitive grooming. Fourth, loss of α_2_δ isoforms differentially affected brain/body ratios in ducky, α_2_δ-1/-3 and α_2_δ-2/-3 double knockout mice (Figure 2): in all three analyzed mouse models juvenile mice showed a disproportionate body to brain growth, reflected as a highly decreased bodyweight together with a moderately reduced brain size. Whereas adult ducky mice displayed even higher brain/body ratios, this effect was normalized in α_2_δ-1/-3 double knockout mice until adulthood. Fifth, although direct comparison of the distinct mouse models was precluded as mice were maintained in different genetic backgrounds (see “Materials and Methods” section), the volumetric analysis demonstrated concurrent effects on specific brain regions caused by deletion of distinct α_2_δ subunits (Figure 4): cerebellum and cortex were significantly reduced when α_2_δ-2 was absent, and lack of α_2_δ-3 specifically elicited a volume decrease in the corpus callosum. Of note, the fact that α_2_δ-1/-3 and α_2_δ-1/-2 double knockout mice, which were both maintained in a mixed 129J × C57BL/6N background, displayed distinct disease phenotypes argues against unspecific effects related to the use of different background strains. Thus, these data suggest that loss of α_2_δ isoforms differentially affects postnatal survival, development, and neurological disease.

### Roles of α_2_δ-2 in the Postnatal Brain and Implications for the Brain Motor Circuitry

Given the importance of α_2_δ subunits in synapse formation and wiring (Pirone et al., 2014; Fell et al., 2016; Risher et al., 2018; Geisler et al., 2019; Schoepf et al., 2019) a reduction of distinct brain regions could be either related to developmental impairment or neurodegenerative processes. Underdevelopment of selective regions of the CNS including the spinal cord, pons, and cerebellum was among the first documented features of young (<P26) α_2_δ-2 mutant ducky mice (Meier, 1968; Brodbeck et al., 2002). Notably, besides decreased cerebellar volume, which is most likely associated with smaller and atrophic Purkinje cells (Meier, 1968; Brodbeck et al., 2002), we additionally observed that cortex size was significantly reduced in adult ducky mice. These data are insofar interesting as no overt cortical abnormalities have been described in the original reports of young ducky mice (Meier, 1968). Moreover, cortical malformations have been previously associated with aberrant α_2_δ-1 signaling (Lau et al., 2017) and neurological disorders including epilepsy and autism (Chen et al., 2015; Fenlon et al., 2015; Hong and Mah, 2015). Because layer formation is largely complete by P7, and synapse formation and maturation stabilize until P21 in mice (Li et al., 2008; Farhy-Tselnicker and Allen, 2018), the cortex thus represents an intriguing model system to address possible neurodegenerative and neurodevelopmental processes in ducky mice.

Analyzing thickness, cell densities, and lamination in cortices of adult (8-week, Figure 5) and juvenile ducky mice (3-weeks, Figure 6) led us to draw several principal observations and conclusions: total cortex thickness and cell densities were not altered in juvenile ducky mice, thus corroborating previous studies detecting no obvious anatomical abnormalities in cortices of young mice (Meier, 1968). Importantly, however, adult ducky mice displayed a reduction in total thickness accompanied by a concomitant increase in cell densities. Moreover, cortical lamination was preserved both in juvenile and adult mice. Altogether, these data suggest a progressive decrease of cortical thickness, rather than atypical neuron migration or developmental retardation. Thus, we hypothesize that cortical thinning is likely associated with a reduction in spacing, which reflects the amount of neuronal and glial cell processes, spines, and synaptic contacts.

Notably, we found that the thickness of layer V was most severely and consistently reduced in somatosensory cortices of adult animals. What might be this finding’s relevance to the neuropathological ducky phenotype? In general, layer V pyramidal cells receive excitatory and inhibitory inputs from cortical and thalamic neurons (Slater et al., 2019), while they project their axons to major subcortical motor systems including thalamic nuclei, striatum, pons, and spinal cord (Thomson and Lamy, 2007; Yu et al., 2008). A previous study showed that α_2_δ-2 mRNA was specifically expressed within regions of the motor circuitry including the spinal cord, as well as GABAergic neurons of the cortex, brainstem, cerebellum, habenula, and nucleus reticularis of the thalamus (Cole et al., 2005), all of which are atrophic in young ducky mice (Meier, 1968). Together, these observations close the loop between the above-mentioned brain regions and the ducky phenotype: young ducky mice exhibit an ataxic gait (>P11) and spike-wave discharges (SWD) associated with aberrant thalamocortical oscillations (Meier, 1968; Barclay et al., 2001). We previously found that the presynaptic expression of α_2_δ-2 modulates synaptic connectivity and the localization of inhibitory postsynaptic receptors (Geisler et al., 2019). Thus, it is tempting to speculate that reduced cortical thickness occurs secondary to aberrant connectivity of excitatory/inhibitory imbalance within the brain motor circuitry, ultimately causing destabilization and loss of synapses.

### CACNA2D as Risk Genes for Autism Spectrum Disorders?

Large brain volumes which normalize until adulthood, as well as altered size of white matter structures, are mentioned as specific traits of autism patients (Vidal et al., 2006; Chen and Van Horn, 2017) and animal models with autistic-like phenotypes (Brodkin, 2007; Kane et al., 2012; Wang et al., 2016). Such disease characteristics are suggested to underlie developmental defects causing an excess number of neurons (Courchesne et al., 2011), increased number of synapses (Tang et al., 2014), and aberrant structural/functional connectivity (Wang et al., 2016). Previous studies showed that dysfunction in autistic-brain circuitry leads to abnormal behavior, comprising impaired reciprocal social interaction, communication deficits, and repetitive behaviors (Kane et al., 2012; Wang et al., 2016). Notably, we exclusively detected abnormal behavior comprising signs of stress, anxiety, and repetitive actions in α_2_δ-2/-3 inter-crosses, whose severity was linked to a gradual decrease of the total amount of α_2_δ-2 and particularly α_2_δ-3 isoforms. The fact that all of the above-mentioned phenotypes demonstrated in the present study share similarities with other mutant mouse models displaying autistic-like characteristics (Peça et al., 2011; Kalueff et al., 2016; Wang et al., 2016), contributes to the accumulating evidence of CACNA2D3 as a potential risk gene for autism spectrum disorders (Iossifov et al., 2012; De Rubeis et al., 2014; Landmann et al., 2018a).

### Future Perspectives

The neurological disease phenotypes described here are likely associated with different roles of α_2_δ isoforms associated with VGCC, NMDAR, and so-far unidentified interaction partners, which will inevitably influence each other. For instance, alterations in synapse differentiation will also affect VGCC expression and function, further influencing neuronal excitability and synaptic transmission (see discussion in Ablinger et al., 2020). Moreover, the partially severe phenotypes of existing single and double knockout mice further complicate the issue of addressing specific neuronal functions. In a nutshell, to study the roles of individual α_2_δ isoforms in the normal and diseased brain will require the development of conditional knockout animal models, which will further aid in assessing autism-like behavior in α_2_δ mutant mice.

## Data Availability Statement

The raw data supporting the conclusions of this article will be made available by the authors, without undue reservation.

## Ethics Statement

The animal study was reviewed and approved by Austrian Federal Ministry of Education, Science and Research.

## Author Contributions

SG designed, performed, and analyzed experiments and wrote the manuscript. AB and CS participated in the experimental work and/or data analysis and/or planning/designing of experiments. CS, AS, and NS contributed analytic tools and/or planning of experiments. GO conceived the study, supervised the project, designed and analyzed experiments, and wrote the manuscript. All authors contributed to the article and approved the submitted version.

## Conflict of Interest

The authors declare that the research was conducted in the absence of any commercial or financial relationships that could be construed as a potential conflict of interest.

## Footnotes

^1^http://www.informatics.jax.org/knockout_mice/deltagen/12_MolBio.html

^2^https://imagej.net/ImageJ
