# Supplementary material for: Phenotypic Characterization and Brain Structure Analysis of Calcium Channel Subunit α2δ-2 Mutant (Ducky) and α2δ Double Knockout Mice
Source: Front Synaptic Neurosci. 2021 Feb 19;13:634412. doi: 10.3389/fnsyn.2021.634412 (PMC7933509; doi:10.3389/fnsyn.2021.634412)
Supplement: Supplementary file 1 [file Data_Sheet_1.PDF]

Supplementary Table 1. List of primary antibodies used for analysis of cortical lamination.

Antibody	Immunogen	Host, type, clone	Dilution	Source
Ctip2	Fusion protein of human CTIP2 (aa 1–150)	rat, monoclonal, 25B6	1:500	Abcam Cat# ab18465, RRID:AB_2064130
Cux1	Mouse Cux1 (CDP) (aa 1111–1332)	rabbit, polyclonal, M-222	1:200	Santa Cruz Biotechnology Cat# sc-13024, RRID:AB_2261231
Tbr1	Synthetic peptide of mouse Tbr1 (aa 50-150) conjugated to keyhole limpet haemocyanin	rabbit, polyclonal	1:500	Abcam Cat# ab31940, RRID:AB_2200219

Supplementary Table 2. Raw data and statistics of brain region volumes of individual mice (Figure 4). *Abbreviations*: Cb = cerebellum, Cc = corpus callosum, He = whole hemisphere, Hc = hippocampus, Nx = neocortex.

	Brain region volume (mm^3)				
**Genotype**	**Cb**	**Cc**	**He**	**Hc**	**Nx**
$\alpha_2\delta$-$2^{+/+}$	16.4	3.4	149.6	8.7	34.9
	22.6	3.4	173.2	8.3	38.6
	18.7	3.0	146.8	8.6	33.2
$\alpha_2\delta$-$2^{du/du}$	13.2	2.9	104.8	6.4	20.0
	10.9	2.2	107.4	8.5	23.7
	16.7	2.5	137.5	6.5	26.3
T-value[a]	2.3	3.25	2.96	2.0	5.03
Raw p-value[b]	0.08	0.03	0.004	0.12	0.007
Corrected p-value[c]	0.17	0.13	0.12	0.12	0.04
$\alpha_2\delta$-$1^{+/-}$, $\alpha_2\delta$-$3^{+/-}$ or $\alpha_2\delta$-$1^{+/-}$, $\alpha_2\delta$-$3^{+/+}$	23.8	4.1	156.1	9.4	33.6
	24.6	4.0	162.4	10.7	36.3
	21.5	4.0	148.5	10.1	33.7
$\alpha_2\delta$-$1^{-/-}$, $\alpha_2\delta$-$3^{-/-}$	17.8	3.0	130.2	8.4	31.2
	19.0	3.1	140.1	8.6	33.2
	21.8	3.1	145.7	9.1	33.7
T-value[a]	2.46	16.47	3.34	2.8	1.59
Raw p-value[b]	0.07	0.00079	0.049	0.03	0.19
Corrected p-value[c]	0.14	0.0003	0.15	0.12	0.19
$\alpha_2\delta$-$1^{-/-}$, $\alpha_2\delta$-$2^{+/+}$ or $\alpha_2\delta$-$1^{-/-}$, $\alpha_2\delta$-$2^{du/+}$	22.0	2.7	171.0	8.2	38.6
	17.1	2.8	137.7	6.7	31.6
	23.1	3.1	146.2	6.0	26.3
$\alpha_2\delta$-$1^{-/-}$, $\alpha_2\delta$-$2^{-/-}$	13.1	2.3	107.6	7.0	25.6
	10.0	2.5	98.0	6.7	25.0
	16.1	2.3	124.5	5.7	26.0
T-value[a]	3.04	3.92	3.29	0.61	1.97
Raw p-value[b]	0.038	0.017	0.03	0.57	0.14
Corrected p-value[c]	0.12	0.09	0.12	0.57	0.27
$\alpha_2\delta$-$2^{+/+}$, $\alpha_2\delta$-$3^{+/-}$ or $\alpha_2\delta$-$2^{+/+}$, $\alpha_2\delta$-$3^{-/-}$	17.9	2.8	136.0	7.0	29.9
	18.5	2.7	142.5	8.1	30.8
	16.8	3.0	134.3	8.8	32.8
$\alpha_2\delta$-$2^{du/du}$, $\alpha_2\delta$-$3^{-/-}$	11.4	1.8	103.8	5.4	23.8
	10.3	2.1	105.5	5.8	25.4
	10.3	2.1	94.5	6.9	23.4
T-value[a]	11.66	6.78	8.61	2.68	6.75
Raw p-value[b]	0.00031	0.0025	0.001	0.06	0.0025
Corrected p-value[c]	0.0015	0.0074	0.004	0.06	0.005

[a]4 degrees of freedom; [b]unpaired t-test; [c]P-values adjusted for Holm-Sidak correction for multiplicity

Supplementary Table 3. Fraction of marker positive cells in cortices of $\alpha_2\delta$ mutant ducky and wildtype mice (Figure 5H and 6H).

	Genotype	^{a}Ctip^{-}/Tbr^{-}/ Höchst^{+}	^{b}Ctip^{+}/Tbr^{+}/ Höchst^{+}	^{c}Ctip^{+}/Tbr^{-}/ Höchst^{+}	^{d}Ctip^{-}/Tbr^{+}/ Höchst^{+}
juvenile	$\alpha_2\delta$-2$^{+/+}$	57.23	16.15	5.35	21.27
		64.76	14.48	14.18	6.58
		43.08	28.46	6.10	22.36
		76.83	10.53	6.34	6.29
	Mean ± SEM	60.47 ± 6.11	17.41 ± 3.35	7.99 ± 1.79	14.13 ± 3.85
	$\alpha_2\delta$-2$^{du/du}$	64.24	15.20	13.00	7.55
		65.63	12.96	3.03	18.37
		36.40	31.30	8.60	23.69
		29.17	36.76	8.40	25.66
	Mean ± SEM	48.86 ± 8.14	24.06 ± 5.10	8.26 ± 1.77	18.82 ± 3.51
	P-value	0.14	0.39	0.97	0.55
adult	$\alpha_2\delta$-2$^{+/+}$	50.35	25.43	17.69	6.53
		48.22	25.01	12.21	14.56
	Mean ± SEM	49.29 ± 1.06	25.22 ± 0.21	14.95 ± 2.74	10.54 ± 4.01
	$\alpha_2\delta$-2$^{du/du}$	70.65	11.12	14.46	3.76
		51.42	20.58	10.28	17.71
	Mean ± SEM	61.04 ± 9.61	15.85 ± 4.73	12.37± 2.09	10.74 ± 6.98
	P-value	0.14	0.23	0.72	0.98

[a]layer I-IV neurons and non-neuronal cells of all layers; [b]layer V-VI neurons; [c]layer V neurons; [d]layer VI neurons
